# Multiple melt bodies fed the AD 2011 eruption of Puyehue-Cordón Caulle, Chile

**DOI:** 10.1038/srep17589

**Published:** 2015-12-02

**Authors:** B. V. Alloway, N. J. G. Pearce, G. Villarosa, V. Outes, P. I. Moreno

**Affiliations:** 1 School of Geography, Environment and Earth Sciences, Victoria University of Wellington, Wellington 6140, New Zealand; 2 Department of Geography & Earth Sciences, Aberystwyth University, SY23 3DB Wales, United Kingdom; 3 INIBIOMA, CONICET-Universidad Nacional del Comahue, Bariloche, Argentina; 4 Department of Ecological Sciences and Millennium Institute of Ecology and Biodiversity, Universidad de Chile, Santiago 7750000, Chile

## Abstract

Within the volcanological community there is a growing awareness that many large- to small-scale, point-source eruptive events can be fed by multiple melt bodies rather than from a single magma reservoir. In this study, glass shard major- and trace-element compositions were determined from tephra systematically sampled from the outset of the Puyehue-Cordón Caulle (PCC) eruption (~1 km^3^) in southern Chile which commenced on June 4^th^, 2011. Three distinct but cogenetic magma bodies were simultaneously tapped during the paroxysmal phase of this eruption. These are readily identified by clear compositional gaps in CaO, and by Sr/Zr and Sr/Y ratios, resulting from dominantly plagioclase extraction at slightly different pressures, with incompatible elements controlled by zircon crystallisation. Our results clearly demonstrate the utility of glass shard major- and trace-element data in defining the contribution of multiple magma bodies to an explosive eruption. The complex spatial association of the PCC fissure zone with the Liquiñe-Ofqui Fault zone was likely an influential factor that impeded the ascent of the parent magma and allowed the formation of discrete melt bodies within the sub-volcanic system that continued to independently fractionate.

At 18.45 UTC on June 4^th^, 2011, an explosive eruption (VEI ≤ 5) of rhyolitic magma^1^,^2^,^3^ began at Puyehue-Cordón Caulle volcanic complex (PCCVC), southern Chile and close to the frontier with Argentina. This PCC eruption was the first within this dominantly dacitic to rhyolitic fissure zone since 1960^4^,^5^,^6^,^7^ (Fig. 1) and was preceded (commencing April 27^th^, 2011) by intensifying seismic swarms located between 2.5 and 5 km depth^1^. The eruption commenced with the development of a sustained Plinian eruption column that ascended to >15 km above the newly formed vent. Ash from this column circum-navigated the southern Hemisphere in less than 14 days and severely disrupted aviation in Argentina, Uruguay, South Africa, Australia, New Zealand and finally in Chile itself. By January 2012, explosive activity had declined from sustained Plinian to intermittent sub-Plinian fountaining, to mixed gas- and ash-jetting punctuated by Vulcanian blasts. This explosive activity was accompanied by synchronous effusion of obsidian lava in a hybrid explosive-effusive eruption^1^,^3^. Throughout the PCC eruption two closely spaced (<200 m), and sometimes partly overlapping, explosive loci (NW and SE vents) were noted^3^. The regular occurrence of synchronous blasts from both vents suggested an advanced degree of connectivity in the shallow subsurface.

The position of the 2011 PCC vent(s) coincides with the intersection of the N-S oriented Liquiñe-Ofqui Fault zone with the 15-km long by 4 km wide NW-SE oriented Cordón Caulle fault-bounded graben with associated fissure vents, aligned domes and pyroclastic cones (Fig. 1).

In this study, we determine the major- and trace-element chemistries of fresh vesicular glass shards using grain-discrete electron microprobe (EMP) and laser ablation inductively coupled plasma-mass spectrometry (LA-ICP-MS) techniques, respectively. Daily ash-fall samples were collected from downwind localities (*SI Table 1*) from the commencement of the PCC eruption on June 4^th^, 2011 in order to quantify any compositional evolution of the rhyolitic magma as the eruption progressed. Fresh vesicular glass was specifically selected during analysis to minimize potential contamination by conduit and/or overburden glassy materials that may have been incorporated during magma ascent.

## Methods

### PCC ash collection and isopach map

**Figure 2:**
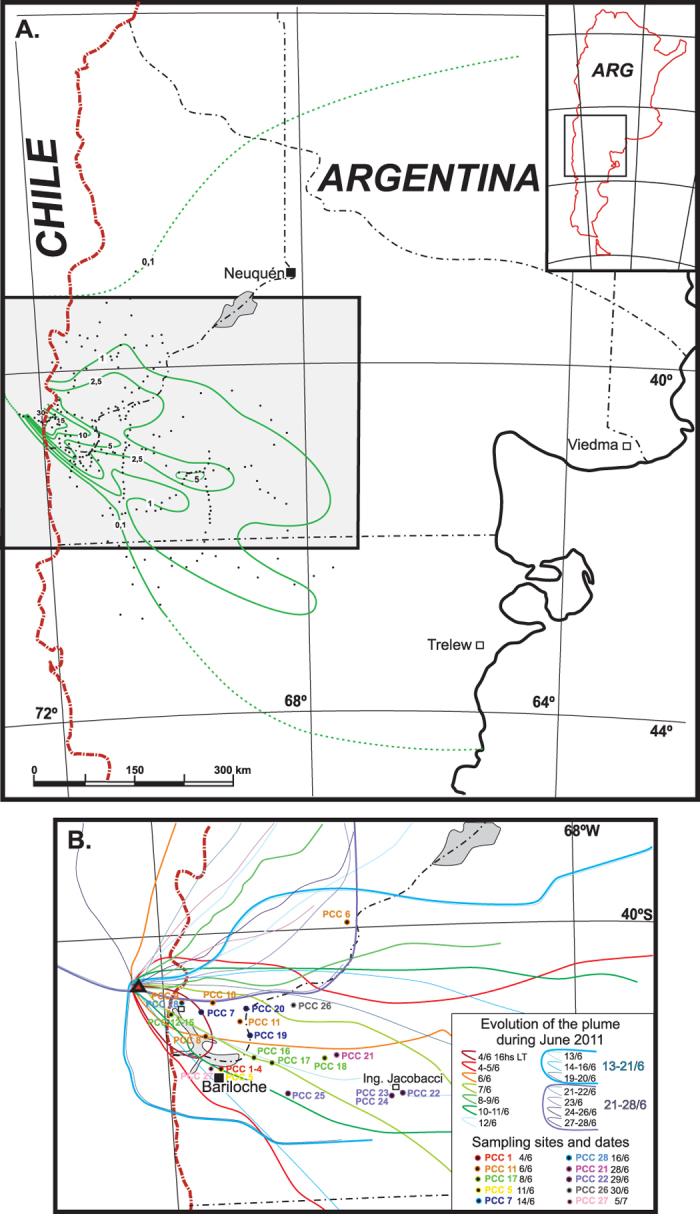
(**A**) Isopach map of the AD 2011 PCC eruption across eastern-most Chile and southern Argentina including Uruguay (modified from *ref*. 9). All ash measurement locations are indicated. Isopachs expressed in centimeters. (**B**) Map showing the evolution of the plume during June 2011. Locations of tephra collection sites are indicated (*see SI Table 1*). Maps were modified and/or drawn using Adobe Illustrator CS5 version 15.0.2.

Ash was systematically measured and collected from planar surfaces and collection receptacles from the outset of the PCC eruption on June 4^th^ following a set of instructions^8^,^9^ adapted from IVHHN and USGS procedures. Based on this extensive collection network of 400 sampling points, which spanned the western half of the Argentine Provinces of Neuquén and Rio Negro, we produced a modified isopach map that indicates total composite ash thickness deposited during the paroxysmal phase of the eruption (Fig. 2A) with a volume of ~1 km^3^ (see also *ref.* 10). We also utilized NASA MODIS satellite imagery taken during the PCC eruption to monitor the plume during June 2011. Our isopach data highlights the complexity of mapping this variably directed and widely dispersed PCC plume by a sustained eruption of relatively small volume (Fig. 2B). On this basis, it is clear that this PCC eruption shares many characteristics with that of the equally small-volume A.D. 2010 Icelandic eruption of Eyjafjallajökull Volcano that created significant disruption across mainland Europe due to its rapid dispersal by prevailing westerly winds^11^,^12^.

### Electron Microprobe (EMP) technique

Major element determinations were made on a JEOL Superprobe (JXA-8230) housed at Victoria University of Wellington, using the ZAF correction method. Analyses were performed with 15 kV accelerating voltage, 8 nA beam current, and an electron beam defocused to between 20 to 10 μm. Standardization was achieved by means of mineral and glass standards. A rhyolitic glass standard (VG-568) was routinely used to monitor calibration in all analytical runs, and used to evaluate any day-to-day differences in the calibration. The large number of samples precluded conducting all analyses in a single batch. All analyses are normalized to 100 wt.% anhydrous, with H_2_O by difference being given, and total Fe is reported as FeO. Glass shard major element analyses are presented in *SI Table 2*.

### Laser ablation inductively coupled plasma-mass spectrometry (LA-ICP-MS) technique

Trace element analyses on individual glass shards were performed by laser ablation (LA) ICP-MS in the Department of Geography and Earth Sciences, Aberystwyth University, using a Coherent GeoLas ArF 193 nm Excimer LA system coupled to a Thermo Finnegan Element 2 sector field ICP-MS. Trace element data were collected for individual shards with the majority of analyses performed using 20 μm ablation craters. Laser fluence was 10 Jcm^−2^ at a repetition rate of 5 Hz for a 24 second acquisition. The minor ^29^Si isotope was used as the internal standard, with SiO_2_ (determined by EMPA) used to calibrate each analysis, after normalization to an anhydrous basis. The NIST 612 reference glass was used for calibration, taking concentrations from *ref.* 13. A fractionation factor was applied to the data to account for analytical bias related to the different matrices of the reference standard and the sample. For this factor as well as ICP-MS and laser operating conditions see *ref.* 14, and references therein. The MPI-DING reference glass ATHO-G^15^ was analysed as an unknown under the same operating conditions at the same time. Analytical precision is typically between ±5–10%, and accuracy is typically around ±5%, when compared with the published GeoReM concentrations for ATHO-G. Glass shard trace element analyses are presented in *SI Table 3*.

### X-ray fluorescence (XRF) and solution inductively coupled plasma mass spectrometry (ICP-MS) techniques

Bulk glass major and trace element compositions of PCC eruptive material were obtained by X-ray fluorescence (XRF) and solution inductively coupled plasma mass spectrometry (ICP-MS) analyses conducted at CRL Energy Ltd. SpectraChem Analytical facilities located in Lower Hutt, New Zealand, and at Victoria University of Wellington, New Zealand, respectively. Washed pumiceous lapilli were crushed and then sieved between 250- to 63-um. Crystal and glass components were then separated using a Frantz magnetic separator, with the purity of the separated glass visually assessed using a binocular microscope. A total of 15 analyses were carried out: one sample (PCC-7) was run in duplicate and the BCR-2 basaltic rock standard was analysed to check accuracy. Bulk glass major (XRF) and trace element (Solution-ICP-MS) were determined using Siemens SRS3000 and Agilent 7500CS ICP-MS instruments, respectively, with samples bracketed by the primary standard (BCR-2). Analyses are presented in *SI Table 4*.

## Geochemistry and Grain-Size Attributes

**Figure 3:**
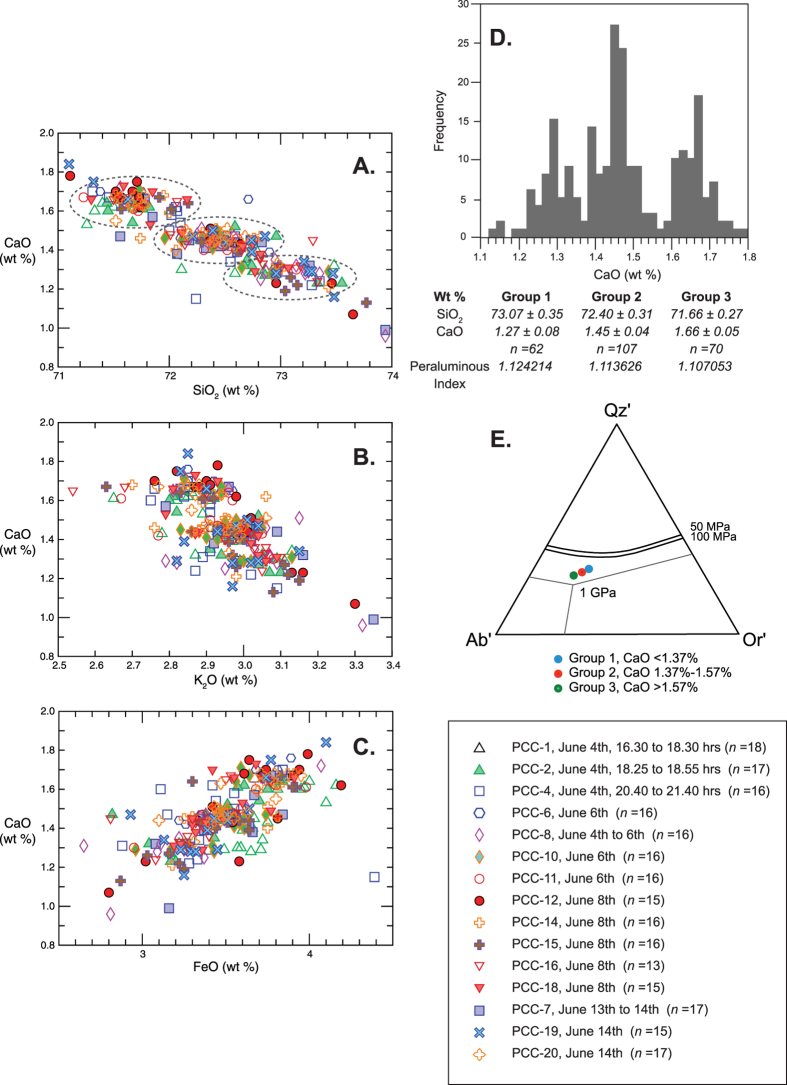
(**A**–**C**) Selected major element compositions (weight percent SiO_2_ vs. CaO and FeO vs. K_2_O and CaO) of glass shards from PCC tephra collected in time series; (**D**) Frequency histogram of glass shard wt% CaO identifying compositional gaps of 1.37 and 1.57 wt% respectively; All PCC-glasses are peraluminous (molar Al_2_O_3_>(Na_2_O+K_2_O+CaO)) and thus corundum normative; (**E**) Projection of the PCC glass compositional groups into the synthetic system Qz’-Ab’-Or’ (see *ref.* 16). Selected H_2_O saturated phase boundaries in Qz-Ab-Or haplogranitic melts as a function of pressure are indicated.

Major element analyses conducted on glass shards reveal three compositional groupings (Fig. 3A–C; *SI Table 2*) defined by minima in CaO concentrations at 1.57 and 1.37 wt% (Fig. 3D). These groups are present in each of the samples collected on different days indicating that they are not analytical artefacts. With increasing SiO_2_, there is a steady decrease in CaO, FeO, MgO, TiO_2_, Al_2_O_3_ and Na_2_O, with a corresponding slight increase in K_2_O, consistent with fractionation of sodic plagioclase, pyroxene and magnetite/ilmenite (cf. *ref.* 2). Projection of average group compositions (divided on CaO, see above) into the synthetic system Qz’-Ab’-Or’ (following *ref.* 16) shows evolution of the magmas towards granite minimum compositions (Fig. 3E), but never reaching a cotectic or minimum composition. The magmatic evolution between the three groups instead suggests ascent-driven crystallisation with three separate pockets of magma becoming arrested and evolving at different depths in the sub-volcanic system and most likely, at pressures of between 100 to 50 MPa (see *ref.* 2, cf. *ref.* 16). Indeed, these experimentally determined crystallisation pressures are in broad agreement with the 2.5- to 5-km depths of pre-eruption seismic swarms that commenced on April 27^th^, 2011.

**Figure 4:**
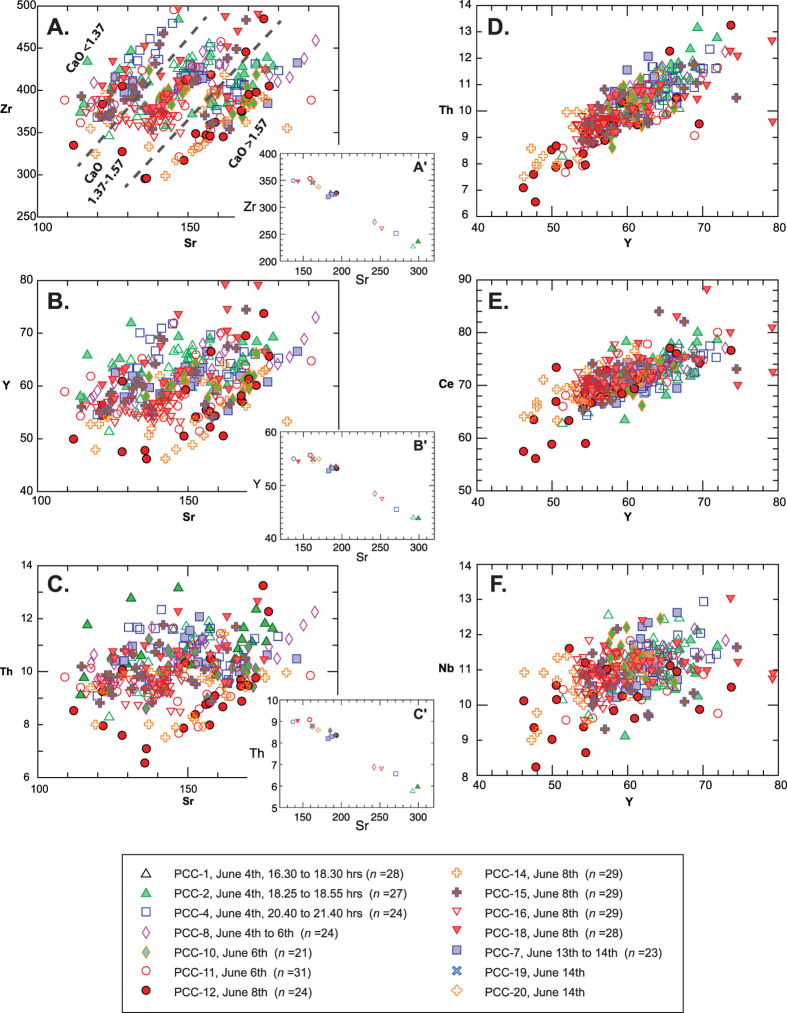
Selected trace element plots of PCC samples. Sr vs. Zr, Y and Th (**A**–**C**) indicate three closely related compositional fields that correspond with major element compositional clusters identified in Fig. 3 (**A**–**C**). The PCC glasses are all peraluminous (corundum normative) rhyolites, containing between 300–400 ppm Zr, and thus will be zircon saturated^23^. The variation of Sr in bulk samples (see insets **A′**–**C′**) indicates that plagioclase fractionation is important in the evolution of the PCC magma, is consistent with both phenocryst observations and experiment (cf. *ref.* 2), and mirrors the major element data. In the glasses, Zr, Y and Th (**A**–**C**) (all highly compatible in zircon) decrease with decreasing Sr indicating the concurrent fractionation of zircon alongside plagioclase. Incompatible elements such as Y vs. Nb, Ce and Th (**D**–**F**) show no variation in element/element ratios between the three magma groups, indicating the cogenetic relationship of the simultaneously erupted magma bodies.

Close inspection of the data shows a gradual decline of CaO with increasing SiO_2_ within each group, superimposed on the general between-group trend of decreasing CaO with increasing SiO_2_. Trace element data (Fig. 4; *SI Table 3*) mirrors these major element trends, with Sr echoing the groupings based on CaO compositions, and Sr/Zr ratios clearly defining the three separate evolving magma batches (with Sr/Zr <0.34, 0.34–0.39, and >0.39). Variations in Zr, Y and Th result from minor zircon extraction within these groups concurrent with dominant plagioclase (and pyroxene) fractionation (Fig. 4A–C). Incompatible elements (including Nb, Y, Zr, REE, Hf, Ta, Th, U) show consistent element/element ratios attesting to the co-magmatic origin of the three magma groups (Fig. 4D–F). Once again, the trace element data (e.g. Sr vs. Zr, Y and Th) shows that each separate magmatic group is represented in each of the individual days’ eruptive products (see Fig. 4), indicating the simultaneous tapping of these compositionally distinct melt bodies.

The bulk chemistry shows a trend of plagioclase extraction that defines a fractionation trend between a phenocryst assemblage dominated by plagioclase and the EPMA glass compositions (72.37 ± 0.60 wt.% SiO_2_; 1.43 ± 0.08 wt.% CaO) *(SI* Fig. 1; *SI Table 4)*. Incompatible trace element ratios are consistent between bulk sample and individual glass shards (particularly when the compatibility of some trace elements in phenocrysts is considered, e.g. Y in pyroxene), and these compare well with similarly acquired data *(SI* Fig. 2) from Puyehue Volcano and its adjacent volcanic massif 7.

Laser auto-analyser grain-size analyses were also conducted on each tephra sample and indicate multimodal grain-size peaks (*SI* Fig. 3), though without conducting grain discrete geochemistry on glass shards from each of these peaks it is difficult to accurately assess the relationship of a particular grain-size component to a potential magmatic source. Irrespective of this, the key control in determining the grain-size characteristics appears to be dominantly influenced by the variable meteorological conditions affecting the ash column at different altitudes and on different days in combination with variations in eruptive flux^10^.

## Discussion

Despite a number of studies^9^,^10^,^17^,^18^ that closely examined the products of the AD 2011 PCC eruption and, indeed, verified its eruptive complexity, the contribution of magma reservoirs and their subtle geochemical differences were not recognised. Our results clearly demonstrate the utility of grain discrete glass shard major- and trace-element data as a relatively simple and straightforward method by which the involvement of multiple magma bodies contributing to a point-source eruption can be easily detected. We suggest that the formation of separate pockets of intermediate to evolved magma, which arrest at different depths during ascent, and then become variously involved in a point-source eruptive event, is likely to be a more common eruptive phenomenon than previously recognised (i.e. see *ref.* 19), especially in highly complex volcano-tectonic environments such as the Andes.

The involvement of multiple magma bodies during the paroxysmal phase of the AD 2011 PCC eruption may also partially explain why hybrid explosive-effusive activities were near-simultaneously produced^3^. Our results are consistent with earlier studies (i.e. *ref.* 6) suggesting that the highly fractured nature of the PCC fissure zone was influential in defining the tempo and style of volcanism within this zone. The complex setting of the PCCVC, adjacent to regional tectonic structures such as the Liquiñe-Ofqui Fault zone, coupled with its extant (pre-2011) magmatic plumbing system, is likely to be influential in allowing or impeding magma ascent, and thus potentially facilitating the formation of separate (independent, fractionating) melt bodies within the sub-volcanic system.

Recent petrological and InSAR measurements^20^ propose a pre-existing laccolith-shaped magma reservoir at 4 to 7 km depth, with an inferred area of 20 km^2^, which may have fed all three historical (1921, 1960 and 2011) eruptions. This extensive reservoir presumably ‘leaked’ magma upwards forming the three compositionally distinct melt bodies involved in the 2011 PCC eruption. How these melt bodies might have been temporally and spatially linked with each other and with this larger reservoir system and then simultaneously activated remains to be determined. The application of intra-crystalline diffusion studies^21^,^22^ should eventually reveal more specific time-series changes in magmatic conditions prior to, during and after magma emplacement while analysis of pre- and syn-eruption InSAR and seismic data along with future geophysical transects across this fissure zone may locate and configure magma storage levels, assess interconnectivity as well as identify spatial relationships between surface and sub-surface fault and pre-2011 magmatic structures. Ultimately, these approaches will be useful in clarifying some of the unresolved questions posed from this research.

## Additional Information

**How to cite this article**: Alloway, B. V. *et al.* Multiple melt bodies fed the AD 2011 eruption of Puyehue-Cordón Caulle, Chile. *Sci. Rep.* **5**, 17589; doi: 10.1038/srep17589 (2015).

## Supplementary Material

Supplementary Information

## Figures and Tables

**Figure 1:**
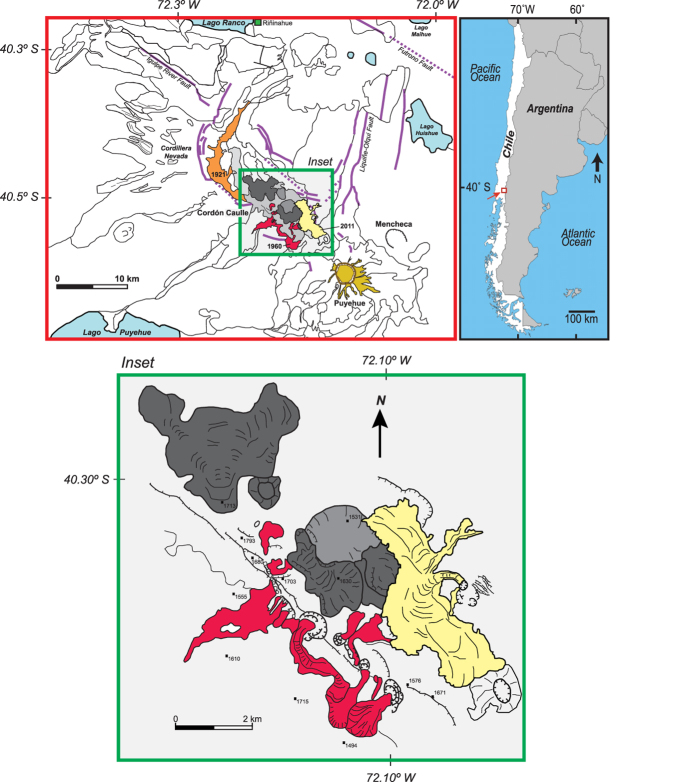
Map of the southeast portion of the PCC volcanic complex showing the location and spatial arrangement of 1921 (coloured orange), 1960 (coloured red) and 2011 (coloured yellow) fissure vents and resultant lava products (inset modified from *ref*. 5). Surveyed points are indicated in meters above sea level. Maps were modified using Adobe Illustrator CS5 version 15.0.2.
