# Supplementary material for: Multiple melt bodies fed the AD 2011 eruption of Puyehue-Cordón Caulle, Chile
Source: Sci Rep. 2015 Dec 2;5:17589. doi: 10.1038/srep17589 (PMC4667254; doi:10.1038/srep17589)
Supplement: Supplementary Information [file srep17589-s1.pdf]

Supplementary Information

Multiple melt bodies fed the AD 2011 eruption of Puyehue-Cordón Caulle, Chile

B.V. Alloway, N.J.G. Pearce, G. Villarosa, V. Outes, and P.I. Moreno

SI Figure Captions:

SI Figure 1. Weight percent SiO_2 vs. FeO/MgO **(A)** and CaO **(B)** concentrations of AD 2011 PCC tephra samples collected in time series determined from bulk glass shard XRF analyses compared with averaged equivalent grain discrete EMP data; (**C, D**)**.** Individual EMP data points for PCC samples collected in time series showing the dispersion of sample data within the overall population cluster. Error bars shown are ± 1 SD.

SI Figure 2. MgO, K_2O, FeO/MgO vs. SiO_2 concentrations determined from bulk glass shard X-ray fluorescence (XRF) analyses conducted on PCC tephra sample splits compared with similarly acquired data from historic to Quaternary-aged Puyehue-Cordón Caulle volcaniclastic materials (see *ref. 7*).

SI Figure 3. Spatial arrangement of laser grain-size distributions of AD 2011 PCC tephra samples collected in time series. Graph colours relate to dates of deposition.

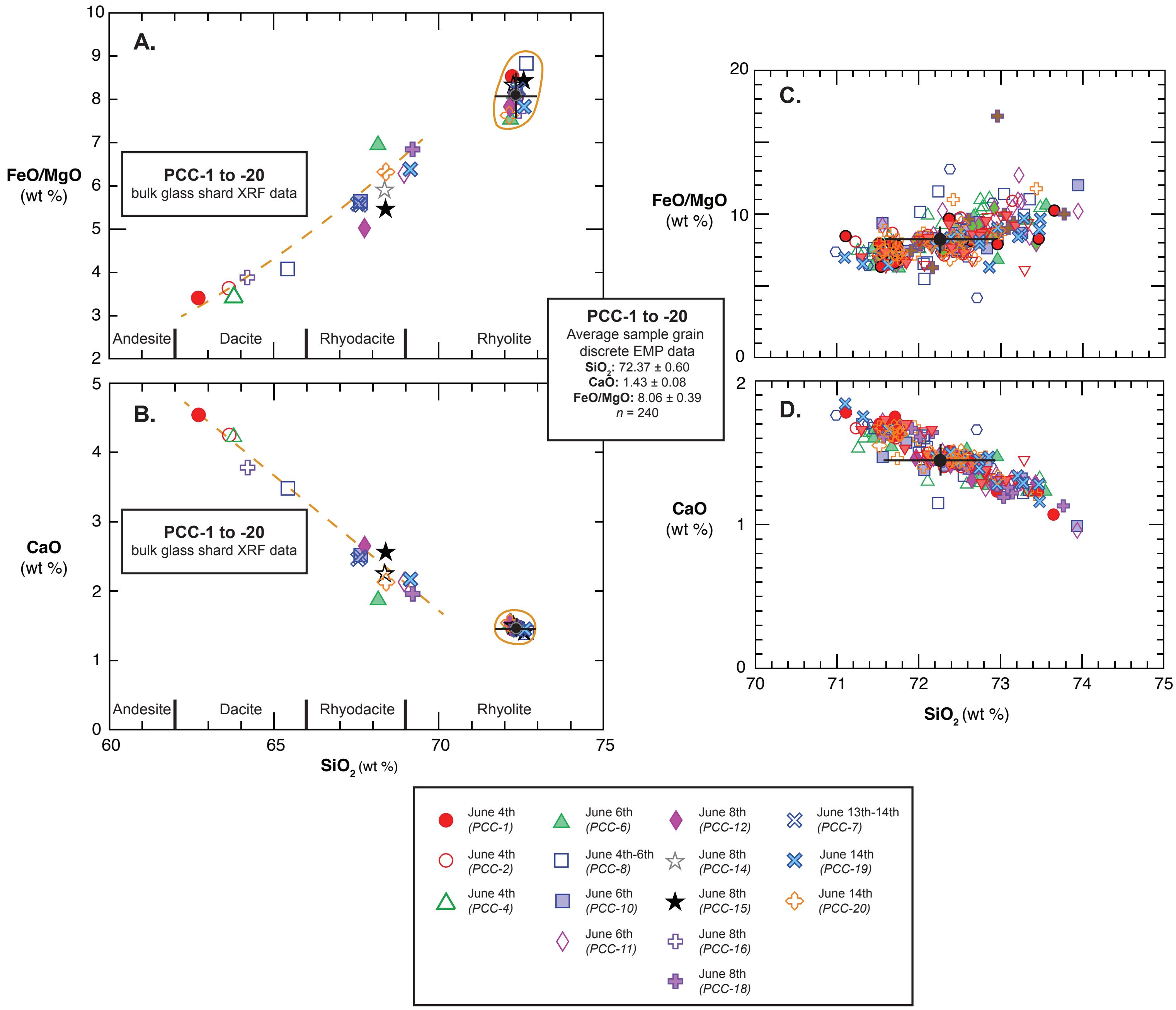
A.
B.
C.
D.
FeO/MgO (wt %)
CaO (wt %)
SiO2 (wt %)
PCC-1 to -20
bulk glass shard XRF data
Andesite
Dacite
Rhyodacite
Rhyolite
PCC-1 to -20
Average sample grain discrete EMP data
SiO2: 72.37 ± 0.60
CaO: 1.43 ± 0.08
FeO/MgO: 8.06 ± 0.39
n = 240
June 4th (PCC-1)
June 4th (PCC-2)
June 4th (PCC-4)
June 6th (PCC-6)
June 4th-6th (PCC-8)
June 6th (PCC-10)
June 6th (PCC-11)
June 8th (PCC-12)
June 8th (PCC-14)
June 8th (PCC-15)
June 8th (PCC-16)
June 8th (PCC-18)
June 13th-14th (PCC-7)
June 14th (PCC-19)
June 14th (PCC-20)

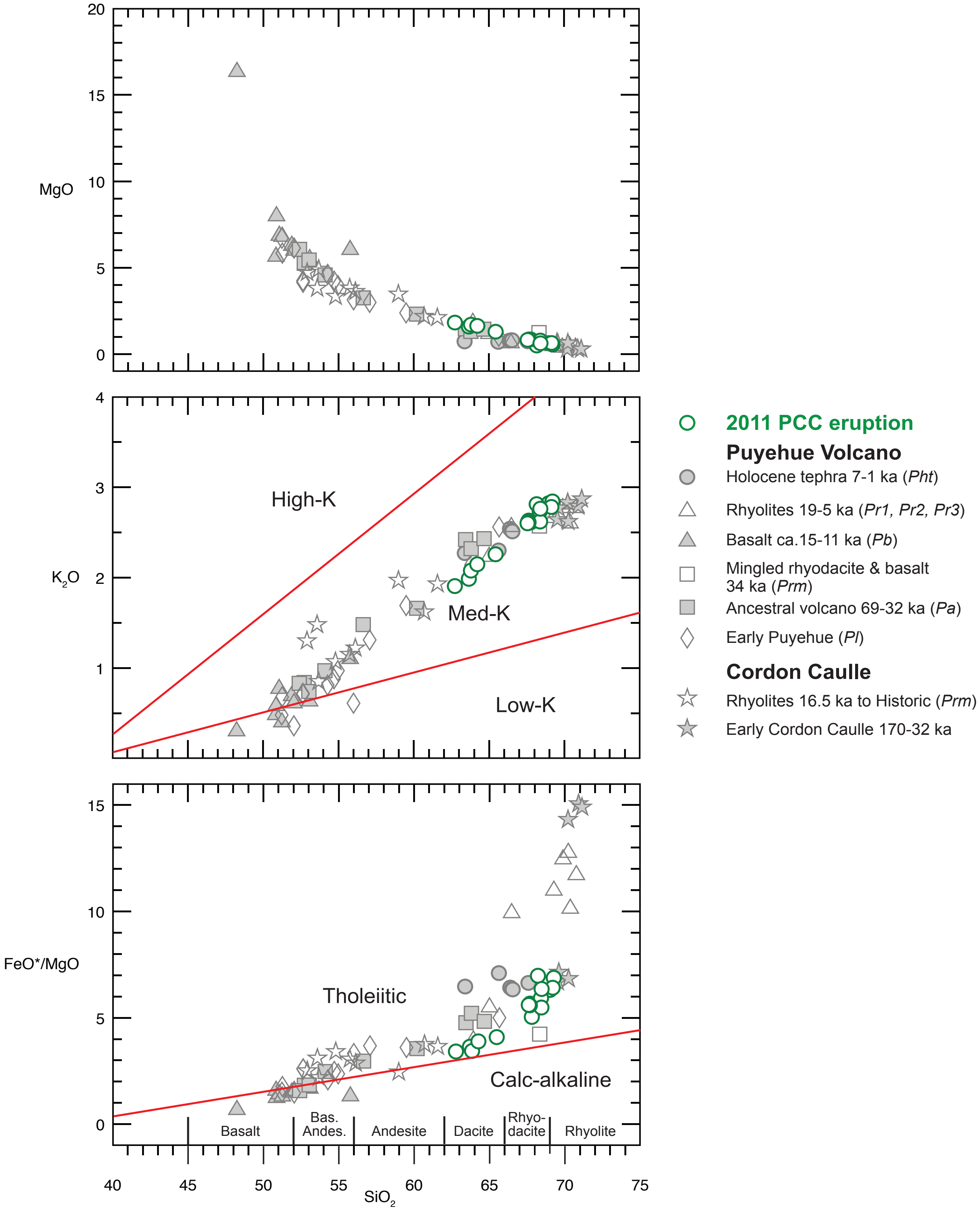

MgO
K_2O
High-K
Med-K
Low-K
FeO*/MgO
Tholeiitic
Calc-alkaline
Basalt
Bas. Andes.
Andesite
Dacite
Rhyo-dacite
Rhyolite
SiO_2
2011 PCC eruption
Puyehue Volcano
Holocene tephra 7-1 ka (Pht)
Rhyolites 19-5 ka (Pr1, Pr2, Pr3)
Basalt ca.15-11 ka (Pb)
Mingled rhyodacite & basalt 34 ka (Prm)
Ancestral volcano 69-32 ka (Pa)
Early Puyehue (Pl)
Cordon Caulle
Rhyolites 16.5 ka to Historic (Prm)
Early Cordon Caulle 170-32 ka

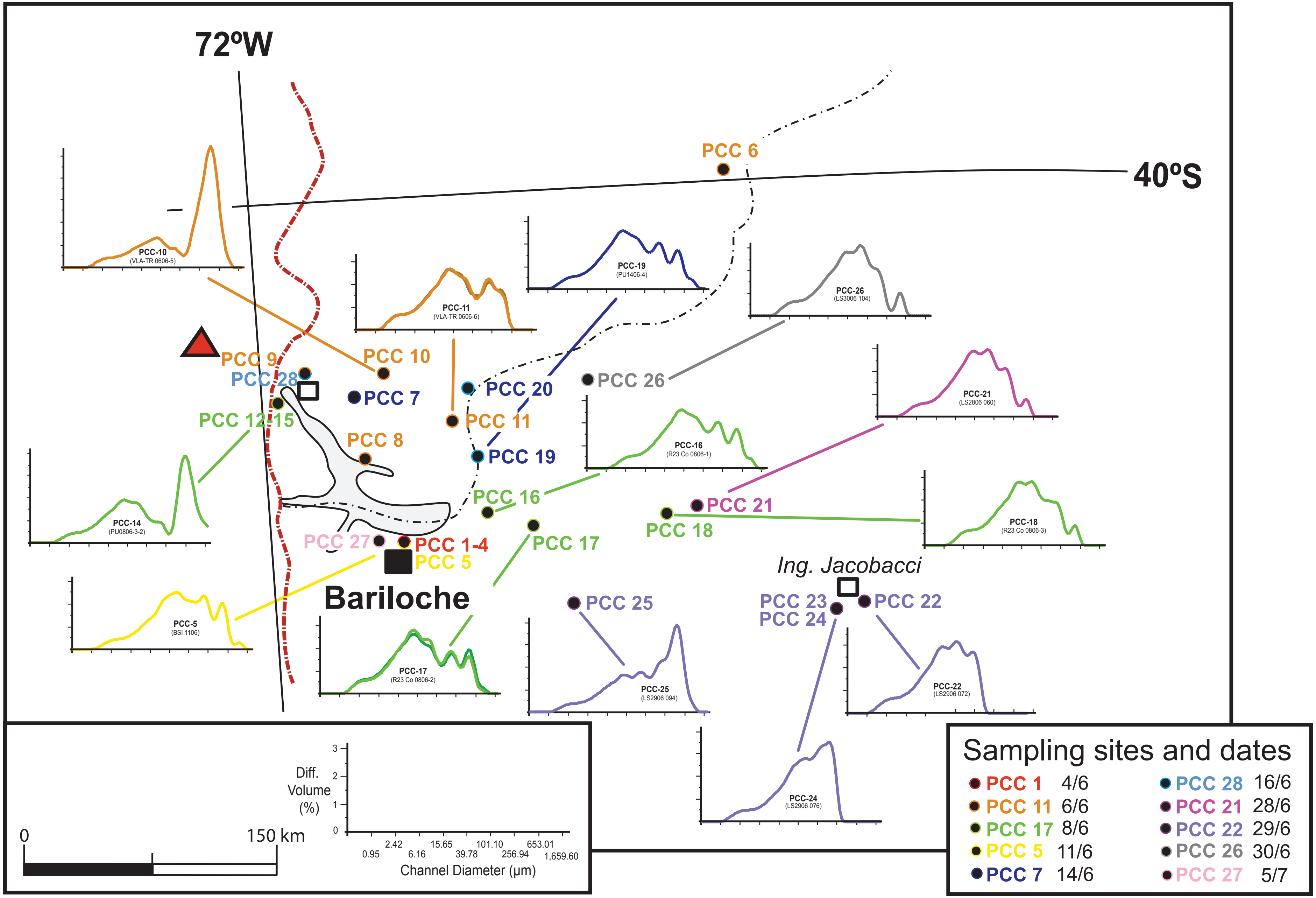

72ºW
40ºS
PCC 6
PCC 9
PCC 28
PCC 10
PCC 7
PCC 20
PCC 26
PCC 12-15
PCC 11
PCC 8
PCC 19
PCC 16
PCC 21
PCC 18
PCC 17
PCC 27
PCC 1-4
PCC 5
Bariloche
Ing. Jacobacci
PCC 25
PCC 23
PCC 24
PCC 22
PCC-10 (VLA-TR 0606-5)
PCC-11 (VLA-TR 0606-6)
PCC-19 (PU1406-4)
PCC-26 (LS3006 104)
PCC-21 (LS2806 060)
PCC-16 (R23 Co 0806-1)
PCC-18 (R23 Co 0806-3)
PCC-14 (PU0806-3-2)
PCC-5 (BSI 1106)
PCC-17 (R23 Co 0806-2)
PCC-25 (LS2906 094)
PCC-22 (LS2906 072)
PCC-24 (LS2906 076)
0
150 km
Diff. Volume (%)
3
2
1
0
0.95
2.42
6.16
15.65
39.78
101.10
256.94
653.01
1,659.60
Channel Diameter (µm)
Sampling sites and dates
PCC 1 4/6
PCC 11 6/6
PCC 17 8/6
PCC 5 11/6
PCC 7 14/6
PCC 28 16/6
PCC 21 28/6
PCC 22 29/6
PCC 26 30/6
PCC 27 5/7

SI Table Captions:

SI Table 1. Locational information of AD 2011 PCC tephra samples collected in time series. Volcanic ash leachate soluble fluorine and pH results are also presented.

SI Table 2. Major element composition of glass shards from the AD 2011 PCC eruption acquired by electron microprobe (EMP) analysis.

SI Table 3. All trace element concentrations in single glass shards from AD 2011 PCC tephra samples obtained by LA-ICP-MS at Aberystwyth. All concentrations in ppm unless otherwise stated.

SI Table 4. Bulk glass shard major (XRF) & trace (Solution-ICP-MS) element compositions of PCC tephra erupted June 4th to 14th 2011.

SI Table 1. Locational information of AD 2011 Puyehue-Cordón Caulle tephra samples collected in time series. Volcanic ash leachate soluble fluorine and pH results are also presented.

PCC	Field designation	Date collected	Comments	Location	GPS coordinates	F** (ppm ash) 1:20	F** (ppm ash) 1:100	pH 1:20
1	BSI 0406-1	**4th June, 2011**	Direct fall sample collected between 16:30 to 18:30 hrs. Samples PCC-1,- 2, -3 and -4 were collected from a plastic bucket during the first pulse of the eruption.	Bariloche (BSI), km 7.600	41° 07' 48.42"S; 71° 24' 6.42"W			
2	BSI 0406-3		Direct fall collected between 18:25 to 18:55 hrs.			13	n.d.	5.45
3	BSI 0406-5		Direct fall collected between 19:31 to 20:01 hrs.			9.2	11	5.89
4	BSI 0406-8		Slightly wet direct fall collected between 20:40 to 21:40 hrs.			9.5	12	5.76
6	R40-PA 0606	**6th June, 2011**	Direct fall collected June 6th.	Piedra del Águila (PA)	40° 03' 06"S; 70 °04' 47.16"W			
8	VLA-TR 0606-3		Samples PCC-8, -9, -10 and -11 were collected during ash fall on June 6th. Sample reflects ash fallen between June 4th to June	Arroyo Ragintuco	40° 54' 0.12"S; 71° 29' 25.20"W	24	31*	5.29
9	VLA-TR 0606-4		Dry sample, very fine ash falling all afternoon.	Lago Espejo Ranger Station	40° 38' 49.38"S; 71° 42' 08.10"W	36	37	5.28
10	VLA-TR 0606-5		Dry sample, very fine ash falling all afternoon.	Villa Traful	40° 39' 12.24"S; 71° 24' 06.84"W	92	83	5.34
11	VLA-TR 0606-6		Sample represents the ash fallen at this site since June 5th. Dry ash falling when sampled.	Población González, La Lipela	40° 47' 29.40"S; 71° 08' 15.54"W	97	107	5.24
12	PU 0806/3-0	**8th June, 2011**	These 4 PCC-samples (PU 0806/3-) correspond to detailed samples through a coarse-grained tephra layer from Villa La Angostura on June 8th. Coarse ash and lapilli at the bottom of the deposit (0-7.5 cm)	Villa La Angostura (near Las Piedritas)	40° 45'47.34"S; 71° 38'24.60"W	12*	n.d.	5.64
13	PU 0806/3-1		Coarse ash and fine lapilli 8 to 10 cm	Villa La Angostura		3.6*	7.1	5.00
14	PU 0806/3-2		Medium to fine ash 10 to 10.8 cm	Villa La Angostura		4.9*	7.7*	5.82
15	PU 0806/3-3		Upper portion of the deposit, coarse ash and lapilli, 10.8 to 11.5 cm	Villa La Angostura				
16	R23Co 0806-1	**8th June, 2011**	Collected on June 8th, slightly wet, sample reflects tephra [illegible]	Perito Moreno	41° 3'30.67"S; 70° 59'51.58"W	9.5*	12	5.82
17	R23Co 0806-2		Collected on June 8th, slightly wet	Pichileufu	41° 05'23"S; 70° 49'43.32"W	11	12	6.21
18	R23Co 0806-3		Collected on June 8th, dry	Comallo	41° 04'10"S; 70° 20'14.34"W	32*	33	5.63
5	BSI 1106	**11th June, 2011**	Direct fall from 11-6, collected before rain fall	loche(BSI), km 7	41° 07' 48.42"S; 71° 24' 6.42"W	34	34	5.5
7	VLARC13_1406	**14th June, 2011**	Direct fall collected between June 13-14th, no rain	Villa La Angostura (VLA), Gendarmería	40° 45'50.82"S; 71° 38'55"W	55	56	5.25
19	PU 1406-4 GPS 019		Fine to medium sandy deposited until June 14th (6-cm thick)	National Route 40, Villa Llanquin	40° 53'47.40"S; 71° 2'28.68"W	22	22	5.92
20	PU 1406-017		Floating pumice in Limay river, collected on June 14th	National Route 40, Confluencia	40° 43'31.32"S; 71° 5'38.58"W			
28	L. Espejo, 160611-2	**16th June, 2011**	Fine sandy ash collected June 16th	Espejo Ranger S	40° 38'49.38"S; 71° 42'08.10"W	94	92	4.85
21	LS2806 060	**28th June, 2011**	Fine sandy ash collected June 28th	Near Comallo	41° 2'56.08"S; 70° 12'39.42"W	73	79	5.17
22	LS2906 072	**29th June, 2011**	Dry sandy ash collected June 29th	Jacobacci aerodrome	41° 19'31.04"S; 69° 34'37.34"W	91	100	5.31
23	LS2906 076a		Coarse sandy basal portion of ash layer collected June 29th,	10 km from Jacobacci	41° 20'29.72"S; 69° 40'21.83"W	13	21	6.85
24	LS2906 076b		Fine sandy upper portion of ash layer collected June 29th	10 km from Jacobacci		43	54	6.61
25	LS2906 094		Fine ash collected June 29th	North of Las Bayas	41° 19'43.93"S; 70° 41'31.96"W	16	24	5.64
26	LS3006 104	**30th June, 2011**	Fine ash collected June 30th	Paso Flores	40° 40'42.82"S; 70° 37'35.65"W	63	61	5.26
27	L. Trébol (0507)	**5th July, 2011**	Collected July 5th, after several rainy days	Laguna El Trébol, km 18 Bariloche	41° 04'28.23"S; 71° 29'22.50"W	4*	10	5.89

Soluble fluorine and pH determinations (Columns G-I) were conducted at Massey University, Palmerston North, New Zealand. Leaching experiments were carried out with ash:solution ratios of 1:20 and 1:100 following the standard methods suggested to aid inter-laboratory collaboration and health hazard analysis (as recommended by the International Volcanic Health Hazards Network (IVHHN) committee on volcanic ash leachate analysis).

**Averages of two replicate extractions with maximum standard deviation of 2 ppm
*No replicates possible due to sample size

SI Table 2. Major element compositions of glass shards from the AD 2011 Puyehue-Cordón Caulle eruption acquired by electron microprobe (EMP) analysis.

Sample	Sample No.	SiO_2	Al_2O_3	TiO_2	FeO	MnO	MgO	CaO	Na_2O	K_2O	Cl	H_2O	*n*
**PCC-1**	PCC-1_1	71.33	14.56	0.61	3.95	0.02	0.54	1.60	4.31	2.91	0.17	0.32	
	PCC-1_2	71.40	14.43	0.54	4.00	0.15	0.57	1.64	4.28	2.86	0.14	-0.79	
	PCC-1_3	72.01	14.42	0.62	3.81	0.03	0.50	1.60	3.98	2.82	0.19	1.16	
	PCC-1_4	72.76	14.02	0.43	3.71	0.00	0.36	1.29	4.13	3.11	0.19	0.08	
	PCC-1_5	72.59	14.19	0.47	3.65	0.04	0.39	1.28	4.15	3.05	0.20	2.33	
	PCC-1_6	72.78	14.13	0.52	3.66	0.07	0.35	1.40	3.95	2.99	0.15	1.60	
	PCC-1_8	72.79	14.03	0.52	3.60	0.00	0.41	1.39	4.02	2.97	0.26	1.38	
	PCC-1_9	72.11	14.09	0.45	3.57	0.08	0.36	1.30	4.77	3.04	0.23	-0.08	
	PCC-1_10	73.48	14.15	0.29	3.22	0.06	0.31	1.32	4.09	2.87	0.21	0.52	
	PCC-1_11	72.69	14.22	0.45	3.72	0.13	0.38	1.34	3.73	3.13	0.22	-0.05	
	PCC-1_12	72.86	14.47	0.46	3.59	0.03	0.32	1.30	3.67	3.09	0.20	1.25	
	PCC-1_13	72.28	14.30	0.52	3.67	0.12	0.40	1.45	4.06	2.99	0.22	0.29	
	PCC-1_14	72.63	14.23	0.50	3.75	0.10	0.44	1.43	3.94	2.78	0.21	0.02	
	PCC-1_16	72.75	14.13	0.44	3.49	0.03	0.32	1.29	4.35	2.99	0.22	-2.34	
	PCC-1_19	71.26	14.55	0.59	4.10	0.06	0.55	1.53	4.30	2.89	0.16	-0.70	
	PCC-1_20	71.64	14.03	0.62	4.16	0.11	0.50	1.62	4.30	2.84	0.19	0.27	
	PCC-1_21	71.68	14.51	0.63	3.97	0.12	0.56	1.61	4.05	2.65	0.22	0.56	
	PCC-1_22	71.50	14.47	0.60	3.78	0.08	0.59	1.62	4.33	2.85	0.18	0.43	
PCC-1 avg.		***72.25***	***14.27***	***0.51***	***3.74***	***0.07***	***0.44***	***1.44***	***4.13***	***2.93***	***0.20***	***0.35***	***18***
s.d.		***0.66***	***0.19***	***0.09***	***0.23***	***0.05***	***0.10***	***0.14***	***0.25***	***0.13***	***0.03***	***1.04***	
**PCC-2**	PCC-2_4	71.67	14.40	0.56	3.70	0.08	0.50	1.54	4.54	2.82	0.18	0.57	
	PCC-2_5	72.38	14.33	0.54	3.45	0.10	0.41	1.44	4.21	2.93	0.20	0.61	
	PCC-2_6	72.59	13.96	0.50	3.51	0.10	0.43	1.52	4.12	3.04	0.22	0.00	
	PCC-2_7	72.70	14.20	0.55	3.43	0.09	0.37	1.35	4.18	2.91	0.21	0.37	
	PCC-2_8	72.96	14.19	0.51	2.82	0.07	0.41	1.47	4.39	2.97	0.21	0.57	
	PCC-2_11	72.48	14.21	0.52	3.38	0.12	0.43	1.46	4.18	3.02	0.20	-0.08	
	PCC-2_12	72.43	14.21	0.53	3.56	0.07	0.38	1.42	4.26	2.96	0.18	0.27	
	PCC-2_13	72.74	14.22	0.46	3.04	0.09	0.38	1.32	4.60	2.94	0.22	0.45	
	PCC-2_14	72.67	14.19	0.45	3.26	0.02	0.33	1.32	4.48	3.05	0.23	0.68	
	PCC-2_15	71.44	14.44	0.58	3.84	0.12	0.60	1.64	4.30	2.82	0.22	-0.36	
	PCC-2_16	71.82	14.38	0.55	3.61	0.10	0.52	1.62	4.42	2.81	0.17	0.07	
	PCC-2_18	71.61	14.44	0.54	3.82	0.14	0.54	1.65	4.18	2.91	0.17	-0.02	
	PCC-2_19	71.76	14.20	0.60	3.57	0.12	0.57	1.61	4.45	2.92	0.18	0.77	
	PCC-2_20	71.45	14.61	0.61	3.75	0.06	0.49	1.60	4.42	2.80	0.20	-0.07	
	PCC-2_21	73.55	13.54	0.48	3.20	0.08	0.30	1.23	4.31	3.07	0.23	0.69	
	PCC-2_22	72.86	14.04	0.46	3.17	0.05	0.37	1.27	4.52	3.04	0.21	0.04	
	PCC-2_23	73.33	13.97	0.49	3.17	0.00	0.33	1.23	4.20	3.10	0.18	0.63	
PCC-2 avg.		***72.38***	***14.21***	***0.53***	***3.43***	***0.08***	***0.43***	***1.45***	***4.34***	***2.95***	***0.20***	***0.30***	***17***
s.d.		***0.65***	***0.24***	***0.05***	***0.29***	***0.04***	***0.09***	***0.15***	***0.15***	***0.10***	***0.02***	***0.35***	
**PCC-4**	PCC-4_1	71.31	14.54	0.62	3.84	0.05	0.53	1.70	4.38	2.85	0.19	0.48	
	PCC-4_2	72.07	14.77	0.59	3.11	0.06	0.57	1.60	4.29	2.75	0.18	1.77	
	PCC-4_5	72.02	14.46	0.55	3.55	0.07	0.35	1.58	4.30	2.91	0.20	0.60	
	PCC-4_6	72.10	14.32	0.58	3.44	0.09	0.52	1.54	4.32	2.91	0.19	0.67	
	PCC-4_8	73.35	14.02	0.44	3.33	0.00	0.30	1.24	4.25	2.87	0.20	-0.90	
	PCC-4_10	71.46	14.62	0.62	3.89	0.09	0.51	1.67	4.16	2.85	0.14	-0.26	
	PCC-4_11	72.55	14.39	0.53	3.34	0.08	0.37	1.34	4.28	2.91	0.21	0.22	
	PCC-4_12	72.59	14.44	0.51	3.60	0.04	0.43	1.47	3.70	3.02	0.19	0.28	
	PCC-4_13	72.51	14.35	0.51	3.15	0.12	0.39	1.47	4.23	3.04	0.23	0.93	
	PCC-4_14	72.00	14.33	0.52	3.55	0.05	0.46	1.50	4.36	3.02	0.21	-0.38	
	PCC-4_15	73.04	14.10	0.47	3.40	0.05	0.30	1.29	4.19	2.99	0.19	-0.41	
	PCC-4_16	72.96	14.36	0.46	2.88	0.02	0.34	1.31	4.58	2.89	0.20	1.04	
	PCC-4_17	72.06	14.59	0.58	3.42	0.03	0.52	1.62	4.19	2.84	0.15	0.65	
	PCC-4_18	72.24	13.53	0.65	4.39	0.10	0.38	1.15	4.27	3.09	0.20	0.19	
	PCC-4_19	73.28	14.06	0.43	3.28	0.01	0.33	1.22	4.22	3.02	0.17	0.05	
	PCC-4_20	71.60	14.34	0.61	3.67	0.09	0.54	1.66	4.52	2.76	0.20	-0.76	
PCC-4 avg.		***72.32***	***14.33***	***0.54***	***3.49***	***0.06***	***0.43***	***1.46***	***4.27***	***2.92***	***0.19***	***0.26***	***16***
s.d.		***0.62***	***0.29***	***0.07***	***0.35***	***0.03***	***0.09***	***0.18***	***0.19***	***0.10***	***0.02***	***0.70***	
**PCC-6**	PCC-6_1	72.94	14.17	0.43	3.28	0.06	0.37	1.30	4.26	3.05	0.14	-0.52	
	PCC-6_2	72.10	14.19	0.54	3.62	0.11	0.45	1.46	4.36	2.96	0.20	-0.57	
	PCC-6_3	70.99	14.48	0.61	3.89	0.12	0.53	1.76	4.54	2.85	0.23	0.04	
	PCC-6_4	72.71	14.63	0.63	2.38	0.03	0.57	1.66	4.29	2.92	0.19	1.66	
	PCC-6_5	72.48	14.23	0.51	3.58	0.09	0.37	1.44	4.35	2.87	0.08	0.00	
	PCC-6_6	71.72	14.54	0.56	3.55	0.09	0.53	1.65	4.22	2.97	0.18	0.84	
	PCC-6_7	72.45	14.14	0.51	3.51	0.10	0.47	1.46	4.26	2.91	0.19	-0.38	
	PCC-6_8	72.49	14.30	0.47	3.26	0.13	0.45	1.42	4.23	3.03	0.22	0.71	
	PCC-6_9	72.90	14.01	0.48	3.38	0.03	0.31	1.39	4.27	3.02	0.21	0.00	
	PCC-6_10	71.75	14.72	0.55	3.68	0.02	0.53	1.70	3.92	2.92	0.21	0.44	
	PCC-6_11	72.58	14.38	0.47	3.22	0.13	0.46	1.44	4.16	2.99	0.18	-0.71	
	PCC-6_12	72.38	14.48	0.58	3.54	0.08	0.27	1.48	4.00	2.99	0.20	0.18	
	PCC-6_13	72.27	14.31	0.50	3.35	0.07	0.46	1.47	4.32	3.04	0.21	0.31	
	PCC-6_14	71.38	14.44	0.60	3.81	0.06	0.60	1.70	4.38	2.85	0.19	0.22	
	PCC-6_15	72.44	14.37	0.47	3.40	0.09	0.46	1.45	4.21	2.94	0.17	-0.36	
	PCC-6_16	71.55	14.39	0.59	3.74	0.03	0.54	1.66	4.38	2.90	0.21	-0.04	
PCC-6 avg.		***72.20***	***14.36***	***0.53***	***3.45***	***0.08***	***0.46***	***1.53***	***4.26***	***2.95***	***0.19***	***0.11***	***16***
s.d.		***0.56***	***0.19***	***0.06***	***0.35***	***0.04***	***0.09***	***0.14***	***0.15***	***0.07***	***0.04***	***0.61***	
**PCC-8**	PCC-8_1	73.14	14.27	0.45	3.11	0.01	0.30	1.32	4.15	3.07	0.18	1.14	
	PCC-8_2	71.64	14.44	0.58	3.74	0.11	0.56	1.65	4.26	2.81	0.21	1.16	
	PCC-8_3	71.56	14.56	0.62	3.72	0.02	0.56	1.66	4.14	2.96	0.21	0.21	
	PCC-8_4	73.21	14.45	0.44	2.65	0.11	0.24	1.31	4.33	3.07	0.21	1.23	
	PCC-8_5	73.25	14.32	0.46	3.18	0.06	0.29	1.29	4.13	2.79	0.23	0.73	
	PCC-8_6	72.29	14.43	0.48	3.62	0.08	0.35	1.51	3.92	3.15	0.15	0.28	
	PCC-8_7	72.58	14.23	0.53	3.38	0.05	0.45	1.47	4.13	2.99	0.19	1.10	
	PCC-8_8	72.25	14.46	0.46	3.51	0.12	0.42	1.48	4.11	3.00	0.20	0.78	
	PCC-8_9	71.56	14.51	0.51	4.07	0.10	0.44	1.72	4.06	2.84	0.20	0.94	
	PCC-8_10	72.95	14.32	0.51	3.22	0.01	0.36	1.35	4.04	3.03	0.21	0.83	
	PCC-8_11	72.87	14.15	0.46	3.25	0.08	0.35	1.29	4.32	3.03	0.20	-0.80	
	PCC-8_12	72.18	14.32	0.51	3.54	0.07	0.41	1.46	4.25	3.06	0.21	0.60	
	PCC-8_13	72.82	14.24	0.46	3.37	0.03	0.38	1.25	4.32	2.95	0.18	-0.54	
	PCC-8_14	73.35	14.11	0.41	3.09	0.06	0.37	1.28	4.29	2.82	0.22	-0.08	
	PCC-8_15	73.22	14.20	0.48	3.17	0.09	0.25	1.28	4.05	3.09	0.18	0.59	
	PCC-8_16	73.94	13.81	0.34	2.81	0.08	0.28	0.96	4.23	3.32	0.24	0.57	
PCC-8 avg.		***72.68***	***14.30***	***0.48***	***3.34***	***0.07***	***0.38***	***1.39***	***4.17***	***3.00***	***0.20***	***0.55***	***16***
s.d.		***0.71***	***0.19***	***0.07***	***0.36***	***0.04***	***0.10***	***0.19***	***0.12***	***0.14***	***0.02***	***0.60***	

**PCC-10**	PCC-10_1	71.69	14.44	0.60	3.56	0.12	0.44	1.64	4.37	2.94	0.18	-0.15	
	PCC-10_2	71.52	14.47	0.64	3.64	0.05	0.52	1.67	4.38	2.94	0.18	0.05	
	PCC-10_3	72.30	14.06	0.53	3.54	0.03	0.48	1.46	4.50	2.93	0.18	0.67	
	PCC-10_4	72.95	14.19	0.47	3.17	0.09	0.37	1.29	4.30	3.00	0.17	-0.79	
	PCC-10_5	72.44	14.39	0.49	3.47	0.06	0.44	1.49	4.17	2.95	0.11	0.02	
	PCC-10_6	71.97	14.20	0.52	3.74	0.05	0.46	1.47	4.38	3.00	0.21	-0.54	
	PCC-10_7	72.43	14.17	0.49	3.51	0.04	0.42	1.50	4.27	2.98	0.21	0.13	
	PCC-10_8	72.39	14.35	0.49	3.55	0.12	0.43	1.41	4.19	2.90	0.19	0.24	
	PCC-10_9	72.73	14.22	0.41	3.61	0.12	0.39	1.40	4.03	2.93	0.16	-0.36	
	PCC-10_10	71.73	14.53	0.60	3.59	0.03	0.56	1.71	4.23	2.84	0.19	-0.13	
	PCC-10_11	72.65	14.00	0.45	3.42	0.04	0.37	1.31	4.37	3.13	0.25	-0.29	
	PCC-10_12	72.52	14.17	0.50	3.58	0.04	0.41	1.43	4.19	2.96	0.19	-0.62	
	PCC-10_13	72.11	14.21	0.57	3.61	0.08	0.44	1.45	4.49	2.83	0.22	-0.38	
	PCC-10_14	73.43	14.06	0.45	2.96	0.09	0.37	1.29	4.20	3.02	0.13	0.41	
	PCC-10_15	71.96	14.20	0.51	3.55	0.13	0.43	1.46	4.50	3.05	0.20	-0.61	
	PCC-10_16	72.92	13.91	0.44	3.43	0.14	0.33	1.28	4.38	2.99	0.20	-0.78	
PCC-10 avg.		***72.36***	***14.22***	***0.51***	***3.50***	***0.08***	***0.43***	***1.45***	***4.31***	***2.96***	***0.18***	***-0.19***	***16***
s.d.		***0.52***	***0.17***	***0.06***	***0.19***	***0.04***	***0.06***	***0.13***	***0.13***	***0.08***	***0.03***	***0.43***	
**PCC-11**	PCC-11_1	72.52	14.40	0.49	3.43	0.06	0.41	1.40	4.10	2.99	0.19	-0.21	
	PCC-11_2	72.40	14.15	0.49	3.35	0.02	0.41	1.43	4.55	3.01	0.18	-0.04	
	PCC-11_3	72.51	14.29	0.53	3.44	0.07	0.35	1.44	4.21	2.94	0.21	0.28	
	PCC-11_4	71.23	14.57	0.61	3.90	0.05	0.48	1.67	4.41	2.90	0.17	-0.63	
	PCC-11_5	71.72	14.41	0.58	3.68	0.05	0.56	1.60	4.23	2.97	0.19	-0.46	
	PCC-11_6	72.40	14.31	0.53	3.43	0.05	0.42	1.47	4.29	2.93	0.17	-0.03	
	PCC-11_7	73.14	14.24	0.45	2.95	0.10	0.27	1.30	4.27	3.08	0.19	0.00	
	PCC-11_8	71.72	14.36	0.61	3.80	0.04	0.51	1.62	4.24	2.91	0.19	-0.57	
	PCC-11_9	71.62	14.67	0.56	3.77	0.08	0.58	1.64	3.93	2.96	0.19	-0.36	
	PCC-11_10	72.31	14.27	0.55	3.39	0.03	0.48	1.40	4.41	2.97	0.19	-0.74	
	PCC-11_11	72.11	14.40	0.45	3.59	0.08	0.42	1.50	4.34	2.91	0.20	-0.34	
	PCC-11_12	72.61	14.09	0.54	3.35	0.07	0.44	1.46	4.28	2.94	0.21	-0.04	
	PCC-11_13	72.47	14.09	0.51	3.35	0.07	0.45	1.43	4.40	3.05	0.18	0.46	
	PCC-11_14	71.68	14.39	0.61	3.98	0.08	0.46	1.61	4.38	2.67	0.14	-0.29	
	PCC-11_15	72.29	14.36	0.53	3.36	0.11	0.45	1.47	4.27	2.99	0.16	-0.57	
	PCC-11_16	72.54	14.27	0.54	3.43	0.11	0.48	1.42	4.28	2.77	0.16	-0.70	
PCC-11 avg.		***72.20***	***14.33***	***0.54***	***3.51***	***0.07***	***0.45***	***1.49***	***4.29***	***2.94***	***0.18***	***-0.27***	***16***
s.d.		***0.49***	***0.15***	***0.05***	***0.26***	***0.03***	***0.07***	***0.11***	***0.14***	***0.10***	***0.02***	***0.35***	
**PCC-12**	PCC-12_1	72.37	14.36	0.51	3.81	0.02	0.39	1.45	3.93	2.98	0.17	0.32	
	PCC-12_2	72.64	14.21	0.52	3.54	0.01	0.44	1.43	4.05	2.98	0.18	0.11	
	PCC-12_3	73.65	14.21	0.38	2.80	0.10	0.27	1.07	3.98	3.30	0.23	0.61	
	PCC-12_4	71.57	14.49	0.59	3.90	0.09	0.54	1.67	4.11	2.87	0.18	-0.75	
	PCC-12_5	71.11	14.84	0.64	3.99	0.12	0.47	1.78	3.94	2.93	0.19	3.78	
	PCC-12_6	71.52	14.16	0.78	3.94	0.08	0.49	1.70	4.36	2.76	0.21	0.19	
	PCC-12_7	71.67	14.57	0.63	3.74	0.10	0.47	1.70	4.01	2.90	0.19	-0.55	
	PCC-12_8	71.73	14.68	0.53	3.72	0.11	0.55	1.67	3.96	2.88	0.18	0.52	
	PCC-12_10	73.46	14.01	0.40	3.02	0.08	0.36	1.23	4.10	3.16	0.19	0.61	
	PCC-12_11	72.96	14.03	0.43	3.58	0.08	0.45	1.23	3.89	3.13	0.21	0.27	
	PCC-12_12	71.54	14.56	0.60	3.61	0.06	0.57	1.68	4.29	2.91	0.18	-0.21	
	PCC-12_13	71.71	14.54	0.64	3.64	0.03	0.55	1.75	4.16	2.82	0.16	0.26	
	PCC-12_14	71.74	14.38	0.53	4.19	0.10	0.54	1.62	3.76	2.98	0.17	3.92	
	PCC-12_15	72.37	14.20	0.55	3.42	0.05	0.43	1.51	4.26	3.02	0.19	0.09	
	PCC-12_16	72.54	14.41	0.50	3.33	0.07	0.41	1.44	4.08	3.01	0.22	0.08	
PCC-12 avg.		***72.17***	***14.38***	***0.55***	***3.61***	***0.07***	***0.46***	***1.53***	***4.06***	***2.97***	***0.19***	***0.62***	***15***
s.d.		***0.76***	***0.24***	***0.10***	***0.37***	***0.03***	***0.08***	***0.21***	***0.16***	***0.14***	***0.02***	***1.37***	
**PCC-14**	PCC-14_1	71.74	14.21	0.70	3.81	0.09	0.47	1.46	4.37	2.93	0.21	-0.58	
	PCC-14_2	72.06	14.01	0.62	3.75	0.14	0.43	1.40	4.41	2.95	0.22	-0.58	
	PCC-14_4	71.59	14.48	0.58	3.89	0.10	0.44	1.63	4.20	2.92	0.18	-0.10	
	PCC-14_5	73.00	14.24	0.43	3.23	0.06	0.37	1.30	4.15	3.05	0.18	-0.46	
	PCC-14_6	72.39	14.29	0.51	3.41	0.13	0.43	1.44	4.24	2.95	0.19	-0.44	
	PCC-14_7	71.95	14.36	0.54	3.87	0.05	0.54	1.68	4.12	2.70	0.18	0.69	
	PCC-14_8	72.30	14.45	0.54	3.50	0.09	0.47	1.49	3.91	3.05	0.19	0.63	
	PCC-14_9	72.76	14.38	0.52	3.30	0.10	0.41	1.46	4.12	2.76	0.19	-0.43	
	PCC-14_10	72.42	14.28	0.50	3.75	0.08	0.34	1.51	4.00	2.95	0.18	-0.12	
	PCC-14_11	72.82	14.24	0.46	3.47	0.07	0.41	1.47	3.91	2.97	0.19	-0.03	
	PCC-14_12	72.38	14.43	0.50	3.45	0.01	0.44	1.48	4.21	2.90	0.20	-0.56	
	PCC-14_13	71.52	14.47	0.61	3.91	0.08	0.49	1.68	4.21	2.85	0.18	-0.66	
	PCC-14_14	73.43	14.12	0.45	3.18	0.10	0.27	1.21	4.06	2.98	0.20	0.66	
	PCC-14_15	72.42	14.28	0.52	3.25	0.11	0.42	1.45	4.49	2.88	0.18	-0.45	
	PCC-14_16	71.66	14.25	0.57	3.82	0.07	0.47	1.62	4.30	3.06	0.17	-0.22	
	PCC-14_17	72.00	14.46	0.58	3.69	0.06	0.45	1.59	4.09	2.89	0.19	-0.46	
PCC-14 avg.		***72.28***	***14.31***	***0.54***	***3.58***	***0.08***	***0.43***	***1.49***	***4.17***	***2.92***	***0.19***	***-0.19***	***16***
s.d.		***0.54***	***0.13***	***0.07***	***0.26***	***0.03***	***0.06***	***0.13***	***0.17***	***0.10***	***0.01***	***0.46***	
**PCC-15**	PCC-15_1	72.37	14.28	0.55	3.58	0.10	0.44	1.44	4.17	2.86	0.21	0.89	
	PCC-15_2	73.15	13.93	0.46	3.22	0.09	0.34	1.22	4.23	3.12	0.22	-0.27	
	PCC-15_3	72.17	14.47	0.60	3.30	0.09	0.53	1.64	4.17	2.83	0.20	0.65	
	PCC-15_4	71.57	14.28	0.61	3.92	0.13	0.54	1.61	4.26	2.89	0.19	0.12	
	PCC-15_5	73.77	13.95	0.46	2.87	0.02	0.29	1.13	4.21	3.08	0.23	0.29	
	PCC-15_6	72.96	14.33	0.47	3.16	0.08	0.19	1.27	4.24	3.11	0.20	1.50	
	PCC-15_7	72.02	14.11	0.57	3.90	0.10	0.50	1.61	4.12	2.92	0.15	0.70	
	PCC-15_8	72.52	14.28	0.50	3.35	0.10	0.44	1.43	4.16	3.03	0.21	0.96	
	PCC-15_9	72.29	14.25	0.53	3.64	0.11	0.45	1.44	4.16	2.97	0.16	-0.01	
	PCC-15_10	73.09	14.26	0.43	3.03	0.03	0.34	1.26	4.38	2.98	0.21	0.65	
	PCC-15_11	72.99	14.06	0.43	3.28	0.04	0.37	1.32	4.35	2.97	0.18	0.16	
	PCC-15_12	73.04	14.05	0.45	3.25	0.07	0.33	1.19	4.28	3.15	0.20	0.15	
	PCC-15_13	71.91	14.27	0.62	3.83	0.06	0.51	1.67	4.29	2.63	0.21	0.12	
	PCC-15_14	72.51	14.27	0.50	3.51	0.05	0.44	1.43	4.13	2.98	0.19	0.04	
	PCC-15_15	72.64	14.26	0.52	3.44	0.09	0.41	1.40	4.04	3.00	0.20	1.11	
	PCC-15_16	72.61	14.19	0.47	3.64	0.06	0.38	1.39	4.12	2.96	0.18	0.40	
PCC-15 avg.		***72.60***	***14.20***	***0.51***	***3.43***	***0.08***	***0.41***	***1.40***	***4.21***	***2.97***	***0.19***	***0.47***	***16***
s.d.		***0.55***	***0.14***	***0.06***	***0.31***	***0.03***	***0.10***	***0.17***	***0.09***	***0.13***	***0.02***	***0.48***	
**PCC-16**	PCC-16_1	71.60	14.57	0.61	3.53	0.12	0.56	1.67	4.49	2.68	0.19	*-0.28*	
	PCC-16_2	72.14	14.54	0.49	3.32	0.06	0.41	1.43	4.38	3.02	0.19	*0.16*	
	PCC-16_3	72.84	14.24	0.48	3.22	0.09	0.33	1.33	4.21	3.06	0.20	*0.17*	
	PCC-16_5	72.65	14.08	0.49	3.37	0.05	0.50	1.40	4.36	2.95	0.16	*-1.73*	
	PCC-16_6	71.50	14.52	0.63	3.71	0.08	0.54	1.66	4.28	2.88	0.20	*-0.03*	
	PCC-16_7	72.07	14.60	0.61	3.50	0.04	0.54	1.65	4.25	2.54	0.19	*0.43*	
	PCC-16_8	71.77	14.52	0.56	3.77	0.04	0.53	1.64	4.08	2.90	0.19	*0.64*	
	PCC-16_9	72.82	14.05	0.51	3.40	0.00	0.39	1.36	4.27	3.03	0.17	*0.09*	
	PCC-16_12	72.81	14.15	0.46	3.27	0.07	0.38	1.36	4.27	3.06	0.16	*-0.23*	
	PCC-16_13	72.01	14.31	0.54	3.56	0.08	0.43	1.48	4.37	3.00	0.23	*1.35*	
	PCC-16_14	73.29	13.99	0.48	2.81	0.08	0.46	1.45	4.35	2.94	0.17	*-2.03*	
	PCC-16_15	73.33	14.11	0.45	3.08	0.04	0.33	1.24	4.18	3.05	0.20	*0.54*	
	PCC-16_16	73.00	13.94	0.43	3.27	0.04	0.33	1.31	4.41	3.10	0.19	*-0.29*	
PCC-15 avg.		***72.45***	***14.28***	***0.52***	***3.37***	***0.06***	***0.44***	***1.46***	***4.30***	***2.94***	***0.19***	***-0.09***	***13***
s.d.		***0.63***	***0.24***	***0.06***	***0.26***	***0.03***	***0.09***	***0.15***	***0.11***	***0.16***	***0.02***	***0.91***	
**PCC-18**	PCC-18_1	73.30	14.11	0.40	3.21	0.01	0.27	1.25	4.12	3.09	0.27	3.48	
	PCC-18_2	73.07	14.15	0.38	3.19	0.06	0.32	1.31	4.25	3.09	0.19	-0.23	
	PCC-18_3	73.18	14.08	0.48	3.23	0.05	0.35	1.32	4.14	3.04	0.14	-0.35	
	PCC-18_4	72.66	14.28	0.50	3.30	0.09	0.38	1.42	4.14	3.04	0.19	-0.16	
	PCC-18_5	72.37	14.41	0.49	3.49	0.10	0.38	1.44	4.34	2.88	0.11	-0.74	
	PCC-18_6	72.16	14.45	0.58	3.55	0.01	0.46	1.66	4.17	2.81	0.14	0.05	
	PCC-18_7	72.77	14.09	0.46	3.36	0.09	0.39	1.29	4.34	3.06	0.16	-0.40	
	PCC-18_8	72.55	14.42	0.43	3.30	0.07	0.45	1.45	4.18	2.99	0.17	0.79	
	PCC-18_9	72.75	14.18	0.55	3.38	0.04	0.40	1.43	4.22	2.87	0.19	0.02	
	PCC-18_10	71.59	14.39	0.59	3.68	0.06	0.50	1.73	4.36	2.87	0.22	-0.46	
	PCC-18_12	71.30	14.42	0.61	3.91	0.11	0.56	1.66	4.41	2.81	0.21	-0.10	
	PCC-18_14	71.83	14.56	0.54	3.60	0.10	0.53	1.53	4.31	2.79	0.20	0.38	
	PCC-18_15	72.11	14.30	0.57	3.77	0.06	0.45	1.49	4.03	3.04	0.19	-0.30	
	PCC-18_16	72.82	14.26	0.47	3.32	0.08	0.35	1.38	4.12	3.02	0.18	0.08	
	PCC-18_17	72.40	14.35	0.45	3.53	0.08	0.46	1.48	4.07	3.00	0.20	0.10	
	PCC-18_18	71.85	14.40	0.56	3.42	0.11	0.52	1.70	4.32	2.93	0.19	-0.76	
PCC-18 avg.		***72.42***	***14.30***	***0.50***	***3.45***	***0.07***	***0.42***	***1.47***	***4.22***	***2.96***	***0.18***	***0.09***	***16***
s.d.		***0.58***	***0.15***	***0.07***	***0.21***	***0.03***	***0.08***	***0.15***	***0.12***	***0.10***	***0.04***	***0.99***	

**PCC-7**	PCC-7_1	73.94	13.57	0.45	3.16	0.07	0.26	0.99	4.02	3.35	0.19	-0.65	
	PCC-7_2	72.49	14.30	0.49	3.30	0.11	0.44	1.44	4.32	2.95	0.15	-0.28	
	PCC-7_3	71.73	14.41	0.61	3.77	0.04	0.56	1.67	4.05	2.96	0.20	0.29	
	PCC-7_4	71.78	14.59	0.53	3.74	0.07	0.55	1.66	4.07	2.83	0.18	-0.01	
	PCC-7_5	72.39	14.43	0.48	3.50	0.04	0.47	1.46	4.06	2.96	0.20	0.64	
	PCC-7_6	72.73	14.20	0.51	3.34	0.08	0.35	1.45	4.16	3.00	0.18	0.19	
	PCC-7_7	72.18	14.26	0.50	3.56	0.09	0.43	1.45	4.38	2.95	0.21	0.04	
	PCC-7_9	71.85	14.42	0.59	3.67	0.08	0.49	1.57	4.38	2.79	0.17	-0.06	
	PCC-7_10	72.49	14.31	0.51	3.42	0.07	0.44	1.45	4.15	2.94	0.21	0.19	
	PCC-7_11	71.56	14.30	0.55	3.84	0.11	0.41	1.47	4.58	3.01	0.17	0.06	
	PCC-7_12	73.26	13.89	0.43	3.08	0.04	0.35	1.32	4.29	3.16	0.17	0.13	
	PCC-7_13	72.58	14.16	0.49	3.57	0.06	0.40	1.44	4.16	2.94	0.20	0.54	
	PCC-7_14	72.33	14.34	0.48	3.64	0.09	0.47	1.41	4.19	2.93	0.13	-0.35	
	PCC-7_15	72.83	14.10	0.55	3.61	0.07	0.47	1.44	3.73	3.09	0.12	0.43	
	PCC-7_16	72.46	14.30	0.54	3.42	0.09	0.39	1.42	4.20	2.99	0.20	0.00	
	PCC-7_17	72.61	14.08	0.52	3.37	0.11	0.42	1.39	4.27	3.02	0.22	0.59	
	PCC-7_18	72.07	14.29	0.49	3.66	0.10	0.44	1.38	4.43	2.92	0.22	0.21	
PCC-7 avg.		***72.43***	***14.23***	***0.51***	***3.51***	***0.08***	***0.43***	***1.43***	***4.20***	***2.99***	***0.18***	***0.12***	*17*
s.d.		***0.59***	***0.23***	***0.05***	***0.21***	***0.02***	***0.07***	***0.15***	***0.20***	***0.13***	***0.03***	***0.34***	
**PCC-19**	PCC-19_1	72.39	14.17	0.52	3.64	0.12	0.44	1.50	4.03	3.01	0.19	-0.63	
	PCC-19_2	72.21	14.21	0.52	3.51	0.04	0.45	1.47	4.45	2.98	0.17	-0.33	
	PCC-19_3	73.47	13.61	0.50	3.29	0.08	0.37	1.28	4.24	2.97	0.19	-0.07	
	PCC-19_4	72.74	14.30	0.50	3.39	0.03	0.43	1.39	4.17	2.84	0.20	0.19	
	PCC-19_5	72.96	14.23	0.48	3.32	0.10	0.37	1.28	4.06	3.02	0.18	0.66	
	PCC-19_6	73.48	14.07	0.40	3.25	0.04	0.34	1.16	4.18	2.97	0.11	0.66	
	PCC-19_7	73.25	13.98	0.43	3.45	0.06	0.40	1.29	4.14	2.82	0.18	0.34	
	PCC-19_8	72.74	14.23	0.50	3.43	0.00	0.42	1.45	4.11	2.93	0.18	0.01	
	PCC-19_9	72.51	14.20	0.46	3.51	0.09	0.41	1.44	4.26	2.93	0.20	0.48	
	PCC-19_10	72.86	14.24	0.52	2.93	0.11	0.46	1.47	4.19	3.04	0.18	0.51	
	PCC-19_11	73.21	14.07	0.43	3.13	0.07	0.37	1.34	4.06	3.15	0.18	0.03	
	PCC-19_12	71.63	14.45	0.61	3.74	0.06	0.58	1.66	4.22	2.90	0.15	-0.16	
	PCC-19_13	71.32	14.67	0.64	3.77	0.07	0.58	1.75	4.18	2.83	0.20	-0.10	
	PCC-19_14	71.10	14.45	0.61	4.10	0.04	0.59	1.84	4.30	2.85	0.12	-0.99	
	PCC-19_15	73.28	14.09	0.45	3.23	0.11	0.33	1.29	3.99	3.04	0.19	0.12	
PCC-19 avg.		***72.61***	***14.20***	***0.50***	***3.45***	***0.07***	***0.44***	***1.44***	***4.17***	***2.95***	***0.18***	***0.05***	*15*
s.d.		***0.76***	***0.24***	***0.07***	***0.29***	***0.03***	***0.08***	***0.19***	***0.12***	***0.09***	***0.03***	***0.46***	
**PCC-20**	PCC-20_2	72.42	14.16	0.62	3.55	0.07	0.42	1.45	4.18	2.96	0.17	-0.74	
	PCC-20_3	71.52	14.38	0.60	3.80	0.14	0.52	1.55	4.46	2.86	0.17	-1.17	
	PCC-20_4	72.38	14.18	0.51	3.10	0.10	0.45	1.44	4.75	2.86	0.22	-0.04	
	PCC-20_5	72.17	14.03	0.55	3.63	0.08	0.45	1.48	4.58	2.98	0.04	0.13	
	PCC-20_6	72.59	14.06	0.53	3.41	0.00	0.44	1.42	4.39	2.99	0.17	-0.44	
	PCC-20_7	72.52	13.99	0.54	3.50	0.09	0.42	1.47	4.30	3.00	0.18	-0.05	
	PCC-20_8	72.52	14.20	0.53	3.43	0.06	0.37	1.51	4.11	3.06	0.22	1.16	
	PCC-20_9	72.97	14.22	0.46	3.31	0.12	0.37	1.33	4.05	2.96	0.20	-0.14	
	PCC-20_10	71.66	14.41	0.58	3.71	0.09	0.54	1.63	4.26	2.94	0.19	0.28	
	PCC-20_11	71.56	14.54	0.59	3.95	0.12	0.53	1.67	4.11	2.77	0.17	-0.72	
	PCC-20_12	71.78	14.27	0.56	3.80	0.05	0.51	1.63	4.39	2.87	0.14	-0.63	
	PCC-20_13	71.76	14.60	0.61	3.47	0.06	0.48	1.63	4.27	2.94	0.19	1.20	
	PCC-20_14	72.50	14.24	0.48	3.47	0.06	0.47	1.44	4.25	2.88	0.21	-0.17	
	PCC-20_15	72.15	14.07	0.52	3.45	0.05	0.44	1.50	4.63	2.96	0.21	-0.04	
	PCC-20_16	71.70	14.41	0.61	3.71	0.03	0.51	1.68	4.33	2.84	0.18	-0.33	
	PCC-20_17	72.68	14.20	0.44	3.47	0.09	0.51	1.47	4.06	2.91	0.17	-0.51	
	PCC-20_18	71.77	14.40	0.65	3.79	0.06	0.55	1.68	4.03	2.88	0.19	-0.24	
PCC-20 avg.		***72.16***	***14.26***	***0.55***	***3.56***	***0.07***	***0.47***	***1.53***	***4.30***	***2.92***	***0.18***	***-0.14***	*17*
s.d.		***0.45***	***0.18***	***0.06***	***0.21***	***0.03***	***0.06***	***0.11***	***0.21***	***0.07***	***0.04***	***0.61***	
PCC-combined	*avg.*	***72.37***	***14.28***	***0.52***	***3.50***	***0.07***	***0.43***	***1.47***	***4.23***	***2.95***	***0.19***	***0.13***	***240***
	s.d.	***0.60***	***0.20***	***0.07***	***0.28***	***0.03***	***0.08***	***0.15***	***0.15***	***0.11***	***0.03***	***0.65***	
**Glass Standard**													
**VG-568**	*avg.*	***76.96***	***12.17***	***0.05***	***1.08***	***0.02***	***0.01***	***0.45***	***3.52***	***4.93***	***0.10***		*46*
25th August, 2013	*s.d.*	***0.61***	***0.10***	***0.02***	***0.20***	***0.02***	***0.01***	***0.05***	***0.23***	***0.12***	***0.03***		

SI Table 3. All trace element concentrations in single glass shards from AD 2011 Puyehue-Cordón Caulle tephra samples obtained by LA-ICP-MS at Aberystwyth. All concentrations in ppm unless otherwise stated.

Sample	Sample No.	Crater diameter um	Int'l std - SiO_2 wt%	Rb	Sr	Y	Zr	Nb	Cs	Ba	La	Ce	Pr	Nd	Sm	Eu	Gd	Tb	Dy	Ho	Er	Tm	Yb	Lu	Hf	Ta	Pb	Th	U	*n*
**PCC-1**	13-19-1-01	*20*	*72.25*	71	150	65.2	427	11.2	3.56	822	37.7	75.3	9.79	42.2	9.85	2.11	10.7	1.39	9.81	2.17	6.86	1.17	6.76	1.33	12.6	0.756	36.1	10.9	3	
	13-19-1-02			72	154	64.8	425	12.4	3.54	956	36.3	74.9	10.5	44.3	9.57	1.56	9.16	1.59	10	2.44	6.73	0.952	7.42	0.971	11.6	0.828	43.4	11.1	2.9	
	13-19-1-03			70.4	157	68.7	455	11	3.56	851	38.4	77.2	11.3	43.7	12.3	1.25	8.86	1.81	10.7	2.79	7.84	1.19	8.82	1.4	13.7	0.691	37	11.9	2.89	
	13-19-1-06			79.9	125	64.1	404	11.5	4.03	800	36.4	71	9.04	37.9	11.2	1.59	9.81	1.62	9.62	2.26	7.67	0.943	6.48	1.49	11.9	0.559	45	10.5	3.27	
	13-19-1-07			74.1	131	60.9	416	11.1	3.98	895	35.3	72.6	9.95	37.1	8.46	1.43	6.7	1.44	9.34	2.22	7.18	1.42	6.68	0.94	10.7	0.725	49	10.1	2.74	
	13-19-1-08			75.1	131	64.9	430	10.5	3.61	801	35.1	72.5	9.53	39.6	9.87	1.12	7.18	1.37	11	2.67	6.85	0.965	6.22	1.2	10.2	0.719	38.1	10.5	2.64	
	13-19-1-10			70.9	150	66	426	11.5	3.72	808	37.8	73.9	9.81	44.1	11.6	1.97	10.3	1.61	9.71	2.37	8.7	1.17	7.72	1.23	12	0.434	40.3	11.5	2.86	
	13-19-1-11			67.2	133	54.8	368	9.7	3.62	871	31.7	64.8	8.45	38.6	8.24	1.37	7.19	1.42	7.92	2.24	6.73	0.917	5.63	0.968	9.52	0.709	37.3	9.19	2.64	
	13-19-1-12			67.8	145	66.5	430	10.7	3.64	789	37.4	74.8	9.52	42.2	11.1	1.76	9.94	1.49	11.2	2.06	8.19	1.15	7.62	1.23	12.3	0.833	40.5	11.6	2.95	
	13-19-1-13			68.7	152	63.7	410	11.5	3.62	876	36.5	71.9	9.73	43.4	8.34	1.1	8.81	1.43	11.2	2.1	7.09	1.14	6.77	1.13	9.21	0.772	38.4	11.4	2.68	
	13-19-1-14			71.8	150	64.1	421	11.4	3.59	805	36.2	73.2	9.8	41.2	9.25	1.65	10.3	1.44	10.5	2.23	7.14	0.969	7.88	0.846	12.2	0.788	44.2	10.9	2.64	
	13-19-1-15			72.4	157	65.6	434	10.9	3.8	853	35.9	76.7	10.9	42.3	8.03	1.62	11.7	1.73	11.2	2.5	6.33	1.32	7.1	1.25	11	0.856	37.3	11.5	2.88	
	13-19-1-16			72.5	147	66.9	425	11.1	3.68	817	36	72.8	9.54	39.9	8.52	1.64	11	1.75	9.76	2.45	8.25	0.934	6.88	1.24	10.5	1.04	37.9	10.1	2.91	
	13-19-1-17			72.2	155	64.8	425	10.6	4.08	812	37.4	76.1	9.58	43	10.7	1.47	12.4	1.87	11.2	2.28	6.92	0.924	8.36	1.32	12.4	0.662	35.2	11.2	3.2	
	13-19-1-18			72.7	151	65.1	430	11.5	4.08	824	36.5	75.6	9.83	43.3	8.52	1.86	10.8	1.7	9.66	2.27	6.79	0.983	7.21	1.2	11.3	0.833	34	11	2.95	
	13-19-1-19			72.1	149	66	430	11.7	3.71	870	37.2	74.9	9.58	40.7	10	1.06	11	1.62	11.8	2.46	8.66	0.936	8.6	1.13	11.6	0.796	43.4	11.7	2.86	
	13-19-1-20			72.6	137	64.8	410	11.7	3.71	785	37.6	69.7	10.2	42.6	11	1.3	11.9	1.67	10.7	2.16	5.86	0.902	7.32	1.38	13	0.742	37.5	10.3	2.99	
	13-19-1-21			70.5	151	67.6	431	11.7	3.5	862	38.6	75.4	9.68	42.4	7.65	1.29	10.8	2.08	11.2	2.22	8.08	1.39	7.1	1.01	10.9	0.735	40.8	11.1	2.88	
	13-19-1-22			66.6	134	57.4	375	12.5	3.99	900	33.7	70.7	9.11	37.9	8.16	1.69	9.1	1.43	10.9	2.28	6.64	1.06	5.79	1.07	11.8	0.706	45.2	10.3	2.84	
	13-19-1-23			66.7	124	51.4	346	10.1	3.32	716	28.8	62.7	8.12	33.2	7.85	1.1	7.01	1.51	8.68	1.92	5.32	0.805	6.03	1.23	9.83	0.699	38.5	8.28	2.89	
	13-19-1-24			67.8	136	56.6	361	11	3.55	829	32.3	70.7	9.19	40.1	10.7	0.882	6.19	1.39	9.53	2.27	8.03	0.529	7.56	0.939	9.41	0.829	34.9	9.16	2.36	
	13-19-1-25			69.6	150	65	437	11	3.82	813	37.2	75.4	10.4	39.1	10.9	1.8	8.76	1.7	10.6	2.43	7.01	1.16	7.68	1.1	10.3	0.803	35.9	10.8	3.09	
	13-19-1-26			72	146	62.5	393	11.4	3.43	800	35.5	71.8	9.05	39.3	10.4	1.83	11.7	1.73	10	2.29	7.36	1.04	6.42	1.28	9.44	0.708	42	9.96	2.99	
	13-19-1-27			73.3	151	66.7	439	11.9	3.56	816	38.4	71	9.45	43.4	7.59	1.04	11	1.72	10.4	2.2	7.12	1	6.98	0.824	11.4	0.39	37.4	10.5	2.49	
	13-19-1-28			76.2	157	66.8	443	12.5	3.66	835	39	77.7	10.7	46.5	10.6	1.43	10	1.7	11.2	2.22	6.8	1.03	8.14	1.35	11.1	0.805	47.2	11.3	2.87	
	13-19-1-31			73	142	57.2	382	11.1	3.35	775	35	67.3	9.51	38.2	8.97	1.3	10.8	1.44	10.7	2.38	7.05	1.06	6.07	1.09	10.7	0.789	43.7	10	3.04	
	13-19-1-32			75.8	144	63.4	399	11.9	3.98	799	34	72	8.73	45.3	7.49	1.37	11.5	1.34	10.5	2.24	6.82	0.978	6.9	1.05	10.5	0.715	41.7	10.8	2.73	
PCC-1 avg.		*All 20 um*	*72.25*	71.7	145	63.4	414	11.3	3.69	829	36	72.7	9.67	41.2	9.52	1.47	9.8	1.59	10.3	2.3	7.19	1.04	7.12	1.15	11.2	0.738	40.1	10.7	2.86	*27*
s.d.				3.09	9.93	4.26	27	0.659	0.214	47.3	2.31	3.58	0.708	2.94	1.41	0.313	1.73	0.18	0.883	0.18	0.792	0.181	0.833	0.173	1.17	0.127	3.99	0.846	0.2	
**PCC-2**	13-19-2-02	*20*	*72.38*	74	115	57.6	383	10.3	4.5	742	32.8	69.3	9.16	38.6	9.69	1.13	10.7	1.65	11.4	2	6.69	1.22	6.55	1.04	9.37	0.925	37.9	9.77	3.01	
	13-19-2-03			75.8	125	63.4	408	11.7	4.29	830	36.2	75.1	9.63	42.1	9.43	1.14	10	1.49	8.42	2.01	6.45	0.939	8.37	1.12	10.7	0.583	38.9	10.6	2.96	
	13-19-2-04			76	130	59.8	402	10.5	4.31	849	35.6	78.3	9.1	40	8.62	1.58	10.1	1.53	7.89	2.32	6.99	0.989	7.46	0.995	10.4	0.757	40.6	10.3	3.32	
	13-19-2-05			84.9	114	58	374	10.6	4.01	793	33.5	77	8.95	39.9	8.79	1.12	8.5	1.43	10.6	2.19	6.6	0.878	5.87	1.06	8.91	0.49	37.6	9.11	2.92	
	13-19-2-06			74.3	117	65.8	434	10.7	3.72	815	36	77.9	10.2	41.7	9.55	1.85	10.6	1.49	11.4	2.35	6.9	1.17	7.13	1.3	10.9	0.89	39.8	11.8	3.18	
	13-19-2-07			72.1	159	59.7	380	10.5	3.51	749	33	69.9	9.41	35.4	8.48	1.69	8.18	1.72	10.2	2.06	6.91	0.911	7.13	1.04	10.6	0.696	37.5	9.87	2.6	
	13-19-2-08			74	151	59.6	372	9.11	3.32	718	33.1	63.4	8.17	37.8	10.6	1.42	10.4	1.42	11.1	1.65	5.79	1.26	6.83	0.99	11.3	0.778	34.3	10.2	2.49	
	13-19-2-10			77.5	157	69	439	10.9	3.91	835	35	72.3	9.58	45.6	8.11	1.32	9.53	1.88	10.5	2.64	6.95	0.89	7.46	1.24	11.1	0.735	40.2	11.6	2.38	
	13-19-2-11			73.1	158	59.1	387	10.3	3.52	769	34.3	71.3	9.64	38.5	9	1.26	9.62	1.46	10.9	1.94	7.32	1.02	7.31	0.983	9.17	0.637	38.3	10.3	2.92	
	13-19-2-12			73.1	143	57.8	358	10.5	3.4	729	32.8	67.1	8.07	37.6	8.19	1.43	9.31	1.26	9.72	1.77	6.22	0.97	5.73	1.05	8.87	0.75	35.6	9.39	2.67	
	13-19-2-13			74.9	167	63.8	415	10.9	3.86	837	35.6	73.2	9.58	37.1	10.2	1.8	10.7	1.62	10	2.24	8.87	0.871	6.71	1.21	10.6	0.862	42.3	10.9	2.95	
	13-19-2-14			82	127	65.1	424	10.8	3.79	795	36.9	68.1	9.72	41	12.4	1.28	10.8	1.58	12.7	2.18	6.27	0.824	8.44	1.36	9.83	0.469	40.4	9.89	3.59	
	13-19-2-15			67.7	173	68.4	432	11	3.41	838	37.2	72.4	9.44	38.2	8.24	1.53	10.2	1.99	10.7	2.47	7.69	1.09	8.03	1.43	12.4	0.755	38.9	12.3	2.68	
	13-19-2-16			71.5	171	66.3	406	10.4	3.7	847	36.9	73.2	10.5	44.5	11.4	1.8	11	1.94	10.7	2.42	6.87	1.32	7.65	1.01	11.4	0.685	38.5	11.1	2.75	
	13-19-2-18			70.3	169	64.5	419	11.4	3.62	786	35.8	73.9	9.91	41.6	10.5	1.59	12.1	1.56	11.2	2.3	5.89	0.942	7.29	1.06	11.7	0.539	36.7	11.6	2.68	
	13-19-2-19			74.2	176	66.9	439	11.6	3.81	833	38.9	75.3	9.94	46.5	10.5	2.09	11.9	2.03	12.2	2.75	7.37	0.997	7.77	1.12	11.5	0.809	37.3	11.1	3.03	
	13-19-2-20			73.3	176	66.8	424	11.6	3.76	835	36.3	74.1	9.58	38.7	8.27	2.01	14.9	1.77	9.83	2.66	8.42	1.04	7.63	1.3	12.2	1.09	38.1	11.8	3.12	
	13-19-2-21			73.1	178	69.2	428	12.1	3.82	833	37.2	78.2	10.3	44.9	9.47	2.05	12.8	1.77	11.2	2.52	7.97	0.972	9.42	1.21	10.7	0.608	35.5	11.6	2.77	
	13-19-2-22			69.4	168	68.3	428	11.8	3.51	797	35.4	71.7	9.36	43.1	10.7	1.72	8.47	2	10.4	2.38	6.93	1.62	7.59	1.38	12.3	0.499	37.7	11.2	3.04	
	13-19-2-23			71.7	166	63.7	404	10.5	3.7	798	35	71.7	9.71	43.4	10.1	2.03	9.11	2.04	11.7	2.59	6.03	0.841	8.09	1.47	12.4	0.651	35.9	10.5	3.08	
	13-19-2-24			73.9	168	65.3	405	11.4	3.76	811	34.1	72.2	9.39	42.5	10.4	1.64	11.3	1.4	11	2.3	7.64	0.866	7.6	1.11	11.8	0.993	37.8	10.5	3.05	
	13-19-2-25			72.9	166	62.8	404	11.4	3.73	805	33.7	73.4	9.77	44.1	11	1.48	11.3	1.18	10.2	2.15	6.04	0.907	5.82	1.04	10.1	0.719	36.6	10.4	2.7	
	13-19-2-26			73.5	163	60.1	384	11.3	3.7	791	34.8	69	9.06	38.1	8.23	1.3	9.15	1.62	10.2	2.21	6.15	0.915	6.54	1.09	10.9	0.801	33.7	9.5	2.98	
	13-19-2-32			73.7	134	62.4	413	10.2	3.83	838	35.8	74	9.75	38.8	9.86	1.8	5.17	1.43	9.42	2.21	7.92	0.985	6.98	1.23	11.5	0.82	39.6	10.6	3.37	
	13-19-2-33			75.8	131	71.9	447	11.7	3.7	859	39.1	78.6	10.1	49.6	10.7	1.87	8.17	2.35	10.6	2.64	6.69	1.07	9.04	1.41	13.2	0.456	37.9	12.8	3.26	
	13-19-2-34			84.7	147	69.3	484	10.2	4.22	882	38.3	80	10.6	52.1	13	1.8	10.6	1.49	12.4	2.14	8.17	1.02	7.89	1.18	12.1	0.479	43.1	13.1	3.32	
	13-19-2-35			72	158	60.7	383	10.8	4.18	770	32.3	69.4	9.7	41.7	10.6	1.41	10.3	1.47	9.08	1.98	6.39	1.11	7.72	1.01	8.98	0.718	41.3	10.6	2.92	
PCC-2 avg.		*All 20 um*	*72.38*	74.4	152	63.9	410	10.9	3.8	807	35.4	73	9.57	41.6	9.85	1.6	10.2	1.65	10.6	2.26	6.97	1.02	7.41	1.17	10.9	0.711	38.2	10.8	2.95	*27*
s.d.				3.99	20.6	4.08	27.7	0.66	0.295	41	1.88	3.92	0.584	3.92	1.29	0.294	1.79	0.274	1.11	0.276	0.815	0.175	0.889	0.151	1.19	0.164	2.25	1.01	0.285	
**PCC-4**	13-19-3-01	*20*	*72.32*	74.3	164	64.3	408	11.6	3.64	731	34.5	69.4	9.47	41	8.46	1.87	9.98	1.57	10	2.15	7.15	0.909	6.42	1.15	11.6	0.737	39.9	10.2	2.91	
	13-19-3-02			69.5	169	62	407	11.1	3.61	760	33.6	69.8	9.43	39.3	9.75	1.51	9.48	1.36	10.6	2.21	7.07	1.05	8.16	0.938	11.1	0.696	37.8	10.1	2.94	
	13-19-3-03			71.6	168	62.5	409	11.7	3.64	745	34.2	68.8	9.15	43	7.78	1.43	7.83	1.72	11.5	2.52	6.4	1	7.16	1.17	10.6	0.912	38.6	10.3	2.98	
	13-19-3-04			71.3	163	62.2	401	11.1	3.56	745	32.6	68.6	9.2	37.7	8.38	1.49	9	1.61	9.66	2.02	7.21	1.09	7.49	0.878	10.2	0.62	36.5	9.91	2.87	
	13-19-3-05			71.7	169	66.5	406	10.5	3.58	776	34.4	71.7	9.75	41.3	10.1	2.29	10.9	1.97	10.8	2.31	6.7	0.904	7.44	1.08	11.9	0.824	38.1	10.8	3.16	
	13-19-3-06			75.3	133	63.4	435	10.7	3.86	774	35.6	73	10.2	42.1	9.56	1.71	10	1.57	10.7	2.46	7.04	0.99	7.05	0.978	11.5	0.576	40.6	11.7	3.13	
	13-19-3-07			78.5	134	70.1	448	12.9	3.94	820	37.6	76	9.78	43.2	10	1.25	11.6	1.52	10.8	2.49	7.62	1.25	8.07	0.886	11.9	0.876	40.7	11.6	3.24	
	13-19-3-08			74.7	130	63.3	437	10.9	3.75	782	35.3	70.7	9.49	40.8	8.85	1.5	10.5	1.44	9.59	2.25	7.73	1.13	7.76	1.2	10.7	0.595	38.5	11.7	3.13	
	13-19-3-09			78.1	145	71.8	479	11.4	3.58	823	37.6	75.3	10.7	46.2	10	1.61	11.6	1.6	11.6	2.93	8.3	0.976	8.8	1.18	12.5	0.971	39.4	11.6	2.92	
	13-19-3-10			73.2	137	68.9	461	11.5	3.79	799	36.6	72.4	10.3	45.2	8.68	1.43	10.5	1.48	10.8	2.69	8.16	1.05	7.54	1.09	11.4	0.877	37.9	10.9	2.84	
	13-19-3-11			79	139	66.7	443	12	4.27	835	36.9	77	10	41.7	10.2	1.69	10.3	1.7	10.9	2.38	7.51	1.03	7.37	1.37	12.2	0.899	40.9	11.6	3.27	
	13-19-3-12			75.3	140	65.1	437	11.2	3.81	831	37.2	75	9.8	46	9.46	1.49	9.6	1.49	10.8	2.37	7.13	1.16	7.42	1.14	13.5	0.731	40.4	10.8	3.1	
	13-19-3-13			72.7	180	65.3	423	11.4	3.72	797	36.1	73.8	9.66	43.7	10.5	1.71	11.4	1.66	11.2	2.29	7.81	1.16	7.47	1.12	10.8	0.726	44.4	11.3	2.96	
	13-19-3-14			70.6	172	66	431	11.2	3.48	797	36.3	74.6	10.1	43.2	10.1	1.72	11.3	1.67	10.7	2.18	6.79	1.06	7.22	1.25	11.8	0.723	38.2	11.2	3.03	
	13-19-3-15			74.8	141	70.9	470	11.3	4.07	851	38.9	77.5	9.92	47.1	10.6	1.61	10.5	1.69	12.5	2.49	7.25	1.2	7.93	1.26	11	0.669	43.4	12.3	3.24	
	13-19-3-17			72.2	164	61.7	392	10.7	3.55	720	32.4	70.2	9.15	39.6	10.5	1.98	9.66	1.5	11.2	2.06	7.24	1.01	7.22	1.07	10.2	0.628	37.6	9.69	2.85	
	13-19-3-18			69.3	159	62.2	400	10.7	3.76	766	34.8	69.8	9.32	39.5	9.14	1.53	10.1	1.64	10.3	2.63	6.76	0.947	6.96	1.24	9.85	0.575	59.5	10.3	2.87	
	13-19-3-19			74.2	166	61.3	398	11	3.64	758	32.4	71.2	9.12	41.5	8.76	1.69	7.78	1.53	9.63	2.24	6.72	0.988	7.07	1.02	9.7	0.692	39.4	10.3	3.15	
	13-19-3-20			68.5	160	59.1	377	10.9	3.55	724	33.2	67.7	9.01	41.3	8.28	2.03	10.2	1.15	9.9	2.29	6.72	0.926	6.67	1.05	10.1	0.698	39	10.3	3.06	
	13-19-3-21			76.7	120	54.6	368	10.4	4.21	676	31.5	64.3	8.46	36.2	8.3	1.18	7.21	1.39	8.37	2.56	6.48	1.03	6.19	0.962	9.68	0.61	44.8	9.09	2.68	
	13-19-3-22			71.7	160	64.6	431	10.9	3.8	790	37.2	73.1	9.91	42.9	9.42	1.95	9.6	1.7	9.77	2.25	7.19	0.892	7.19	1.14	9.84	0.833	42.2	10.6	3.31	
	13-19-3-23			70.1	149	62.7	420	11.7	3.83	781	34.7	70.9	9.22	40.6	9.3	1.56	9.39	1.54	10.8	2.29	7.01	1.08	7.6	1.1	11.7	0.702	39.5	10.9	2.98	
	13-19-3-24			72.2	151	62.7	427	11.3	3.83	763	34.9	72.3	10.3	42.8	9.43	1.4	9.68	1.65	10.1	2.2	7.28	0.958	7.34	1.27	12.4	0.715	40.8	11.2	3.05	
	13-19-3-25			73.4	158	69.7	452	12	3.65	801	38.7	75.6	9.93	43.5	9.17	1.58	12.4	1.97	12.3	2.29	6.56	1.28	8.38	1.17	12.6	0.68	43	11.6	2.91	
PCC-4 avg.		*All 20 um*	*72.38*	73.3	153	64.5	423	11.3	3.75	777	35.3	72	9.64	42.1	9.37	1.63	10	1.59	10.6	2.36	7.16	1.04	7.41	1.11	11.2	0.732	40.9	10.8	3.02	*25*
s.d.				2.9	15.7	3.95	28	0.563	0.204	41.2	2.07	3.22	0.51	2.63	0.804	0.254	1.26	0.175	0.907	0.209	0.501	0.108	0.588	0.126	1.05	0.113	4.53	0.78	0.159	
**PCC-8**	13-19-5-01	*20*	*72.68*	79.7	135	60.2	407	10.7	4.04	731	32.1	70.1	9.39	39.2	8.35	1.43	7.61	1.57	9.18	2.08	6.71	1.12	6.88	1.03	11.7	0.721	44.7	10.2	3.07	
	13-19-5-02			77.4	145	60.5	394	10.7	3.94	786	34	72.6	8.87	36.8	8.9	1.4	9.76	1.46	10.4	2.07	6.33	1.12	7.03	1.04	10.8	0.724	43.7	10.4	3.36	
	13-19-5-03			78.6	153	61.3	413	11.4	3.79	781	35.9	72.3	9.74	42.2	9.89	1.42	10.5	1.64	9.57	2.28	7.7	1.09	6.93	1.1	11.6	0.723	43.8	11	3.18	
	13-19-5-04			74.8	147	59.6	404	11.4	3.92	760	33.7	70.8	9.74	40.7	10.4	1.37	9.45	1.45	10.1	2.32	6.64	0.83	7.36	1.18	11.8	0.745	44	10.4	3.3	
	13-19-5-05			69.9	125	57	381	10.3	3.68	706	30.9	66.8	8.63	37.7	7.7	1.22	10.1	1.57	9.99	1.77	6.14	0.931	6.91	0.922	11.6	0.712	37.6	9.59	3.27	
	13-19-5-06			75.7	132	56.8	381	11.4	3.72	737	32.1	68.2	8.91	38.3	8.62	1.45	7.76	1.57	9.01	1.9	5.41	0.958	6.83	0.92	9.88	0.597	48.5	9.37	3.2	
	13-19-5-07			77	189	68.9	438	12.2	3.77	878	39.6	76.4	10.7	48.9	9.78	1.87	11.9	1.67	13.3	2.53	8.77	1.19	8.45	0.982	11.8	0.857	43.7	11.8	2.9	
	13-19-5-08			76	192	73	459	11.8	3.71	876	39.1	77.1	9.99	51.1	11.5	1.96	10.4	1.7	11.7	2.48	8.58	1.18	8.59	1.26	13.7	0.872	42.1	12.2	2.88	
	13-19-5-09			76.8	158	63.1	408	11	4.04	774	35.1	71.1	9.74	43	8.11	1.67	11.6	1.66	9.63	2.29	7.29	1.06	7.2	0.908	11.2	0.739	45	10.2	3.06	
	13-19-5-10			72.7	180	64.2	410	11.1	3.82	786	33.8	72.4	9.5	47.1	10.9	1.83	10.8	2.08	10.8	2.26	8.44	1.22	7.55	1.09	12	0.773	43.5	10.5	3.28	
	13-19-5-11			76.1	184	65.5	415	11.4	4.16	828	37.5	75.8	9.65	45.3	9	2.22	11.2	2	10	2.23	7.65	0.998	8.41	1.1	12.5	0.915	41.8	11.2	3.27	
	13-19-5-12			81.3	138	64.6	435	11.7	4.42	830	36	73.5	9.95	40.5	11.8	1.61	10.7	1.58	9.56	2.34	6.82	1.08	7.99	0.978	11.4	0.791	45	11.7	3.49	
	13-19-5-14			73.4	161	69.8	452	11.4	4.06	825	37.8	74.4	10	43.5	10.5	1.63	11.1	1.56	10.6	2.47	7.54	1.31	8.25	1.18	12.2	0.838	41.8	11.2	3.02	
	13-19-5-15			75.2	156	65.6	433	10.6	3.71	832	36	74.6	9.95	42.6	10.1	1.62	9.79	1.85	11	2.54	7.66	1.29	8.07	1.29	12	0.707	41.4	11.1	3.37	
	13-19-5-16			72	177	63.9	418	11	3.76	790	37	72	9.21	43.6	10.2	2	11.3	1.63	10.5	2.25	7	1.21	7.32	1.18	11.3	0.657	41.9	10.9	3.14	
	13-19-5-17			71	155	68	446	11	3.89	772	34.8	72.7	10.4	45.1	9.98	1.88	9.51	2.07	10.4	2.35	7.31	1.14	8.31	1.19	11.5	0.721	40.6	11.3	2.83	
	13-19-5-19			72.2	162	66.7	430	11.5	3.8	821	36.4	74.4	10.2	45.3	9.72	1.77	10.8	1.69	11.4	2.29	6.2	1.09	9.06	1.14	11.4	0.866	39.6	11.1	2.88	
	13-19-5-21			73.7	160	65.5	431	11.6	3.77	815	37.1	72.8	10.5	41.7	10.7	1.74	10.6	1.75	11.8	2.67	6.99	1.22	7.29	1.27	11.9	0.703	39	9.99	2.82	
	13-19-5-22			79.6	119	58.1	409	10.7	3.94	768	33.9	72.2	9	41.5	7.56	1.58	8.54	1.76	9.56	1.99	7.11	1	7.17	1.16	12.3	0.763	41.5	10.6	3.36	
	13-19-5-23			82.4	131	60.4	421	10.8	3.82	825	37.1	72.4	9.65	43.3	9.03	1.62	9.18	1.45	10	2.16	7.15	1	7.36	1.25	10.4	0.621	42.1	10.9	4.64	
	13-19-5-24			78.3	118	57.6	385	11	3.94	745	32.7	71	9.55	36.4	8.11	1.43	9.08	1.4	9.4	2.05	6.63	0.92	6.63	1	10.6	0.682	45.3	10.3	3.18	
	13-19-5-25			76.7	120	59.1	386	10.6	4.08	746	32.2	71.5	8.67	34.6	9.07	1.4	8.34	1.58	9.55	2.14	6.58	0.875	6.39	1.08	11.3	0.61	45.2	9.73	3.23	
	13-19-5-26			73.9	127	55.3	367	9.98	3.98	717	31.4	67.5	8.67	37	7.79	1.2	11	1.08	8.84	1.91	5.65	0.91	6.13	1.12	9.18	0.761	40	9.65	3.03	
	13-19-5-27			80.7	125	60.6	416	11.1	4.12	765	34.9	72.2	8.88	39.6	8.48	1.25	10	1.56	10.1	2.09	7.26	1.08	7.28	1.23	10.2	0.718	45.7	10.3	3.56	
PCC-8 avg.		*All 20 um*	*72.68*	76	150	62.7	414	11.1	3.91	787	35.1	72.3	9.56	41.9	9.43	1.61	10	1.64	10.3	2.23	7.06	1.08	7.48	1.11	11.4	0.742	42.8	10.7	3.22	*24*
s.d.				3.34	23.1	4.58	23.8	0.508	0.178	46.2	2.42	2.54	0.596	4.08	1.21	0.264	1.18	0.218	1.02	0.223	0.841	0.132	0.754	0.116	0.94	0.083	2.48	0.734	0.364	
**PCC-10**	13-19-6-01	*20*	*72.36*	75.4	173	66.2	419	10.6	3.9	831	37.5	73.6	10	48.5	8.37	1.8	12.5	1.95	9.77	2.26	8.54	0.919	7.37	1.4	9.97	0.573	42.6	10.1	2.82	
	13-19-6-02			74.6	141	57.8	392	11.1	3.93	733	32.5	68.5	9.64	36.4	9.67	1.26	10	1.34	10.2	2.09	6.94	1.06	6.53	1.28	11.6	0.611	41.2	10	2.93	
	13-19-6-03			72.1	165	57.5	372	11.8	3.67	724	34.3	68.5	8.77	37.6	10.7	1.26	8.5	1.73	10.8	2.25	6.16	1.04	6.77	1.06	10.1	0.629	41.3	10.1	3.05	
	13-19-6-04			76.3	143	60	401	10.8	4.08	803	34.1	72	9.06	40.6	9.52	1.68	11	1.35	10.8	2.05	6.93	0.977	6.3	1.27	10.7	0.696	45.1	10.2	3.12	
	13-19-6-05			72.2	170	59.5	391	11.1	3.71	799	35.1	72.7	10.2	44.8	10.1	1.76	9.18	1.59	9.47	2.59	9.12	1.09	7.55	1.23	9.7	0.802	44.3	10.9	3.02	
	13-19-6-06			73	169	61.2	387	11.5	3.85	757	34.4	70.6	9.57	38	10.7	1.49	9.53	1.53	10.6	2.2	5.99	0.913	6.77	1.07	9.65	0.563	41.3	10.1	2.93	
	13-19-6-07			72.7	168	58.9	368	11.4	3.96	754	33.5	68.6	8.8	40	10	1.31	10.1	1.4	9.34	2.69	7.3	0.925	8.24	1.1	11.8	0.797	43.4	9.72	2.84	
	13-19-6-08			78	137	61.7	424	11.1	4.12	785	36.8	71.5	9.47	42.7	9.82	1.32	10.5	1.64	9.36	2.15	8.49	1.18	8.65	1.03	13.1	0.605	44.9	11.2	3.29	
	13-19-6-10			76.1	149	60.7	402	11.5	4.18	761	36	68.4	9.11	40.6	9.3	1.64	8.5	1.38	9.87	1.88	7.07	1.33	7.35	1.17	9.84	0.739	39.9	10.5	3.09	
	13-19-6-11			74	170	60.7	403	11.2	3.89	774	34.6	69.5	9.59	46.5	10.7	1.85	10.4	1.44	9.77	2.04	6.25	0.954	7.3	1.29	9.99	0.727	43.2	9.98	3	
	13-19-6-12			69.1	157	57.7	390	11.3	3.43	760	32.8	68.4	8.62	39.5	10.5	1.69	10.1	1.5	9.88	2.08	6.82	1.05	7.62	1.07	10.3	0.735	40.8	8.99	2.96	
	13-19-6-15			73.2	145	60.2	383	10.9	3.87	788	34.1	69.3	9.38	39.6	8	1.26	7.97	1.35	9.3	2.04	6.69	0.905	6.76	1.24	10.9	0.609	42	10.6	2.65	
	13-19-6-16			77	142	57.9	397	12	3.69	776	33.7	68.6	9.4	39.4	10.6	1.52	8.58	1.77	9.86	2.26	6.53	1.24	6.9	1.27	9.81	0.614	41.4	9.85	3	
	13-19-6-17			74.6	136	58	380	10.7	3.94	756	33.1	68.9	9.13	37.3	9.14	1.7	8.45	1.51	7.3	2.05	6.12	1.1	6.8	0.938	11	0.618	42.1	8.61	3.13	
	13-19-6-18			75.7	149	61	408	11.1	3.87	775	35.2	69.8	9.01	39.2	10.5	1.44	12.2	1.36	8.1	2.32	5.56	1.11	8.35	1.15	9.77	0.737	43.3	10.4	2.99	
	13-19-6-19			77.5	148	62.4	397	10.7	4.47	827	34.4	73.1	9.05	40	7.8	1.51	8.31	1.51	11	2.32	6.69	1.14	7.82	0.915	9.82	0.795	43.3	10.5	2.83	
	13-19-6-20			76.9	143	59.1	389	11.7	4.03	791	34.2	71.8	9.6	42	7.66	1.69	9.17	1.65	10.3	2.03	7.56	1.02	7.69	1.25	10.3	0.615	47.2	10.8	3.31	
	13-19-6-21			78.7	152	64.3	412	12.4	4.67	835	37.1	74.5	9.53	36.7	8.82	1.67	11.3	1.85	9.42	2.33	7.64	1.09	8.2	0.998	11.2	0.697	47.1	10.8	2.77	
	13-19-6-23			72.2	160	59.8	397	12.2	3.64	752	34.3	68.9	9.17	38	8.64	2.3	7.21	1.59	10.4	2.52	6.94	1.06	7.57	1.19	10.6	0.911	39.9	9.88	2.89	
	13-19-6-24			76.4	170	62.2	385	11.3	4.02	814	34.4	71.9	9.48	43.8	8.88	1.92	9.72	1.63	10.3	2.2	7.26	1.31	6.99	1.03	9.74	0.782	41	10.2	3.19	
	13-19-6-26			73.5	172	61.9	385	11.4	4.38	856	34.9	66.2	10	41.8	8.98	1.5	9.33	1.64	10.5	2.18	7.32	1.06	6.77	1.07	9.73	0.805	46.5	9.44	2.84	
PCC-10 avg.		*All 20 um*	*72.36*	74.7	155	60.4	394	11.3	3.97	783	34.6	70.2	9.36	40.6	9.45	1.6	9.65	1.56	9.83	2.22	7.04	1.07	7.35	1.14	10.5	0.698	42.9	10.1	2.98	*21*
s.d.				2.41	12.8	2.25	14.2	0.49	0.288	34.9	1.32	2.15	0.415	3.2	1.01	0.258	1.37	0.172	0.885	0.199	0.884	0.121	0.651	0.13	0.894	0.0955	2.22	0.624	0.167	

**PCC-11**	13-20-1-02	*20*	*72.2*	76.7	164	68.9	386	11	3.84	777	38.1	91.1	12.8	53.5	14.9	1.54	11.6	1.78	12.4	2.55	7.75	1.04	7.75	1.22	9.99	0.657	37.8	9.07	2.78	
	13-20-1-03			74.6	142	60.2	390	10.6	4.05	800	32.6	70.5	9.48	37.5	7.83	1.52	10.3	1.51	8.76	2.02	6.2	0.835	6.25	1.02	9.35	0.718	41.1	9.57	3.06	
	13-20-1-05			76.7	135	65.2	424	10.4	4.4	808	34.8	70.6	8.8	44.4	10.7	1.27	12.4	1.59	10.2	2.25	7.21	1.07	6.65	1.24	10.9	0.825	39.3	9.87	2.78	
	13-20-1-06			78.6	153	65.9	427	11.4	4.06	849	36.9	74	9.41	42.2	11	1.9	9.75	1.58	11	2.41	7.56	0.843	7.55	1.47	12	0.747	40.8	10.3	2.89	
	13-20-1-07			76.5	142	57.9	393	10.8	3.6	794	33.5	68	8.6	39.5	8.95	1.85	8.36	1.85	9.09	2.22	5.83	1.03	7.25	1.09	9.83	0.806	38.1	9.36	2.94	
	13-20-1-08			80.9	169	63.9	390	11.5	4.19	801	34.4	71.8	9.3	38.6	8.64	1.66	11.2	1.89	9.25	1.85	7.08	0.845	5.82	1.22	10.9	0.519	42.6	9.64	2.71	
	13-20-1-09			72.6	161	57.2	368	11.5	3.4	785	32.5	69.8	9.04	37	8.72	1.49	10.3	1.52	10.1	1.86	6.26	1.23	6.73	0.91	10.7	0.692	40.5	9.26	2.9	
	13-20-1-11			75.8	149	58.5	382	11.5	4.02	768	33.3	69.5	8.56	42.2	9.65	1.93	10.5	1.74	9.75	2.34	7.03	1.21	8.5	0.884	11.8	0.803	40.3	9.81	2.92	
	13-20-1-13			84.4	109	58.9	388	11.5	4.18	811	32	70.9	8.62	35.8	8.98	1.24	8.63	1.3	9.29	1.75	6.35	1.04	5.5	1.11	10.5	0.697	42.1	9.79	2.97	
	13-20-1-14			77	118	61.9	380	10.1	3.55	774	31.8	70.3	8.62	35.6	8.44	1.45	9.72	1.52	9.53	1.93	6.61	0.802	6.62	0.94	9.4	0.656	40.1	10.1	2.81	
	13-20-1-15			80.5	132	58.8	410	11.3	3.86	820	34.7	76.1	9.85	40.7	9.97	1.32	9.73	1.4	9.75	2.36	6.82	0.954	7.5	1	12.3	0.793	44.8	10.7	3.64	
	13-20-1-16			79.3	125	58	383	9.91	4.39	806	31.8	72.4	8.88	39.9	8.78	1.69	9.1	1.5	7.34	2.17	5.72	0.662	6.36	1.04	10.6	0.701	39.2	9.62	3.02	
	13-20-1-17			83.6	121	55.4	370	10.4	4.23	817	31.5	71.7	9.12	40.7	8.08	1.06	8.61	1.34	9.09	1.91	5.16	0.778	6.58	0.95	9.5	0.813	41.3	9.76	2.74	
	13-20-1-18			77.4	114	54.3	362	10.9	4.31	744	30.7	66.8	8.49	34.8	9.91	1.25	6.87	1.39	10.2	2.18	5.96	0.945	6.13	1.07	8.62	0.536	40	9.4	3.3	
	13-20-1-19			81.9	166	60.3	383	11.8	4.22	833	36.3	72.8	9.23	43.2	7.22	1.99	7.77	1.47	9.14	2.35	6.41	1.13	7.22	1.55	11.8	0.533	43.2	10.2	3.53	
	13-20-1-20			76.3	138	55.2	357	10.3	3.9	742	30	67.8	9.33	31.9	8.07	1.46	7.72	1.34	10.7	1.81	6.16	0.75	6.12	0.737	9.17	0.713	39.2	8.88	2.9	
	13-20-1-21			78.1	140	56	364	11	3.73	761	32.2	70.8	8.83	34.8	8.58	1.83	8.48	1.19	9.5	2.03	6.85	1.19	6.3	1	10.3	0.728	39	9.19	3.28	
	13-20-1-22			81.9	145	72	496	9.76	4.03	893	41.1	80.1	10.6	44.8	13.5	3.31	12.4	1.67	11.8	2.5	6.78	1.06	7.73	1.33	11.1	0.74	43	11.6	2.91	
	13-20-1-23			73.3	191	64.8	388	10.7	3.88	860	34.9	72.1	10.2	44.3	11	1.45	6.96	1.54	9.55	2.2	6.21	1	7.19	1.4	8.95	0.56	40.6	9.86	2.92	
	13-20-1-24			78.2	137	55.1	357	11.2	3.91	740	30.8	67.2	8.34	35.2	9.35	1.15	7.91	1.14	8.84	1.88	6.45	0.803	6.53	0.992	9.79	0.636	40.5	9.03	2.53	
	13-20-1-25			75.8	157	56	353	10.3	3.59	749	31.8	68.1	8.91	34.1	7.11	1.17	9.42	1.4	8.46	1.96	5.64	0.974	5.89	0.83	9.32	0.691	41.1	8.84	2.71	
	13-20-1-26			76.5	147	56.2	369	10.9	4.08	798	31.7	71.3	9.06	36.7	9.43	1.49	6.53	1.37	9.24	1.81	6.54	1.09	5.77	0.871	9.08	0.681	36.8	8.88	2.68	
	13-20-1-27			75	147	51.8	321	9.57	3.61	777	29.7	68.1	8.35	36.2	8.73	1.37	6.96	1.43	9.08	1.76	5.34	0.929	6.1	0.912	9.01	0.679	33	7.68	2.46	
	13-20-1-28			77.7	161	70.1	458	10.9	3.71	849	35	74.8	10	46.1	11.4	1.71	13.2	1.44	10.1	3.11	6.49	1.03	8.27	1.03	13.5	0.458	39.9	11.4	2.34	
	13-20-1-29			76.8	126	54.7	366	10.2	3.59	746	31	70.1	8.58	31.4	7.24	1.77	9.54	1.78	8.63	1.62	6.09	0.931	6.71	1.23	9.79	0.502	41.7	9.25	3.13	
	13-20-1-30			74.6	142	54.1	357	11.2	4.33	776	30.7	66.9	8.78	39.4	8.88	1.2	8.87	1.49	9.03	1.7	5.48	0.843	6.76	1.02	10.1	0.483	41	9.24	2.87	
	13-20-1-31			88	151	62.7	428	11.7	3.87	882	37.2	77.8	10.1	42.9	13.8	2.17	7.74	1.84	10.1	2.6	5.51	1.09	7.87	1.09	12.4	1	38	11	3.18	
	13-20-1-32			73.3	157	54.6	343	10.4	4.09	751	31.7	66.1	9.08	35.7	9.43	1.84	7.95	1.4	8.1	1.98	6.1	0.771	5.13	0.987	8.44	0.579	39.3	8.78	2.93	
	13-20-1-33			71.2	154	51.4	341	10.4	3.66	721	29.8	66	8.33	37.3	8.42	1.27	9.02	1.34	7.94	1.93	5.49	0.866	5.49	1.15	10.7	0.757	37.6	8.59	2.97	
	13-20-1-34			74.4	153	54.1	333	10.7	3.69	739	31.2	66.2	8.16	33.6	6.54	1.51	8.24	1.56	8.93	1.63	6	1.01	5.98	1.03	8.48	0.801	37.6	7.92	2.83	
	13-20-1-36			74.9	156	55.3	345	9.63	3.73	759	30.9	68.4	8.68	33.4	9.79	1.33	8.13	1.31	9.06	2.05	6.58	0.945	6.33	0.915	10.7	0.837	37.8	9.15	2.89	
PCC-11 avg.		*All 20 um*	*72.20*	77.5	145	59	381	10.8	3.93	791	33	71.2	9.16	38.8	9.45	1.59	9.16	1.5	9.48	2.09	6.31	0.959	6.66	1.07	10.3	0.688	39.9	9.54	2.92	*31*
s.d.				3.69	17.7	5.36	36.8	0.625	0.276	44	2.71	5.03	0.899	4.86	1.93	0.425	1.71	0.191	1.05	0.331	0.639	0.144	0.841	0.188	1.27	0.123	2.27	0.88	0.274	
**PCC-12**	13-20-2-02	*20*	*72.17*	53.3	136	47.8	296	8.24	2.74	1040	27.5	56.1	7.32	33.3	7.41	1.37	5.85	1.19	9.17	1.48	4.51	0.65	5.39	0.523	5.59	0.223	48	6.56	2.2	
	13-20-2-04			74	112	50	335	9.03	3.95	716	28.1	58.9	8.06	34.2	8.72	1.44	7.46	1.4	7.12	2.03	7.36	0.543	5.03	0.987	8.57	0.589	43.7	8.53	2.75	
	13-20-2-07			77.3	169	69.5	445	9.88	3.88	854	38.6	74.2	10.1	52.5	13.6	0.523	7.76	1.15	13.6	2.23	6.41	1.08	7.75	1.01	12.1	0.895	35.2	9.51	2.73	
	13-20-2-09			73.3	156	55.5	347	10.2	4.05	778	32.5	70.7	8.95	32.1	7.39	1.16	10.5	1.47	9.64	2.2	6.53	1.05	6.36	0.907	8.44	0.852	47.5	8.77	2.8	
	13-20-2-11			87.9	128	60.9	405	9.62	4.03	779	33	69.4	9.58	39	9.4	1.91	8.03	1.64	9.65	2.23	5.79	1.24	8.03	1.24	9.36	0.881	44.7	10.1	2.76	
	13-20-2-12			73.3	122	56.4	384	11.1	4.13	764	31.6	67.8	8.6	35.9	8.59	1.23	6.87	1.37	9.17	1.81	5.09	1.05	7.86	1.12	10.8	0.58	41.2	9.25	2.8	
	13-20-2-13			75.2	153	54.2	349	9.38	3.79	733	29.8	65.6	8.58	36.2	9.13	1.82	8.28	1.5	9.1	1.62	5.74	0.856	6.06	1.05	7.16	0.537	40.7	8.38	2.62	
	13-20-2-14			71.9	157	55.2	359	10.9	3.48	753	30.3	66.7	8.43	34.3	8.53	1.38	9.33	1.44	11.9	1.84	6.47	0.863	5.39	1.02	9.93	0.499	39.9	8.83	2.64	
	13-20-2-15			73.4	168	57	375	11	3.64	783	33.3	68.7	8.74	37.1	9.98	1.55	8.2	1.4	9.33	1.89	6.41	1.11	6.91	1.08	8.92	0.794	43.6	8.89	3.01	
	13-20-2-16			71.5	177	65.6	405	11.1	3.74	863	35	77.1	10.4	42.9	13.4	1.77	16.1	1.59	11.4	2.68	8.74	1.38	8.62	1.11	12.1	0.716	44.1	12.3	3.91	
	13-20-2-17			75.6	170	61.3	395	10.2	4.06	815	34.4	71.2	9.11	38	10.1	1.91	9.37	1.75	10.5	1.97	7.18	0.819	6.49	0.941	9.73	0.631	48.8	9.48	2.93	
	13-20-2-18			71	168	57.2	375	11	3.96	826	33.4	72.8	8.98	39.6	7.65	1.68	8.76	1.54	9.31	2.13	5.82	1.33	6.26	1.03	9.72	0.77	40.6	9.69	2.72	
	13-20-2-19			73.5	173	60.1	398	10.5	3.71	812	35.5	71.9	9.76	41.1	8.41	1.83	9.8	1.61	8.57	2.15	6.13	1.19	6.31	0.981	10.6	0.838	37.6	9.77	2.52	
	13-20-2-21			73.4	159	54.4	362	11.2	3.58	760	31.9	68.6	8.84	33.1	8.2	1.77	9.39	1.7	8.54	1.91	5.67	0.764	6.31	0.956	9.22	0.584	37	9.09	2.6	
	13-20-2-22			73.4	168	58.1	376	9.84	3.81	791	33.3	69.9	9.03	42	10.1	1.44	10.1	1.76	8.69	2.34	6.73	0.863	6.6	0.78	8.51	0.766	37.2	9.46	2.73	
	13-20-2-23			68.1	157	52.2	346	11.6	4.27	738	30.9	63.4	8.69	33.9	7.43	1.46	9.16	1.01	8.71	1.96	5.76	0.803	8.04	0.912	9.53	0.44	38.4	7.99	2.86	
	13-20-2-24			71.3	162	50.6	345	10.2	3.2	860	30.8	73.4	8.7	36.9	9.23	0.936	8.36	1.57	9.21	1.88	6.97	0.793	6.09	0.815	9.14	0.721	47.3	8.68	2.84	
	13-20-2-25			80.3	149	59.3	412	10.3	4.11	805	35.1	68.4	9.71	38.8	8.34	1.58	5.81	1.41	13.3	2.56	6.22	1.28	7.49	1.36	10.9	0.838	41	10.3	2.45	
	13-20-2-26			79	175	73.7	485	10.5	3.66	925	43.2	76.6	10.8	43.8	15.7	1.32	16	2.02	11.5	2.65	9.16	1.09	8.73	1.57	12	0.738	38.8	13.2	4.08	
	13-20-2-27			83.2	158	66.5	418	11	4.17	827	35.9	76	10.5	43.6	9.6	1.93	6.7	1.54	11.5	2.13	8.72	0.585	9.83	1.52	12	0.832	42.4	10.5	2.91	
	13-20-2-28			68.1	128	47.6	327	9.36	3.46	703	29.1	63.5	8.44	34.9	6.48	1.87	6.3	1	8.37	1.77	5.58	0.833	4.45	1.21	7.76	0.504	43.6	7.6	2.99	
	13-20-2-29			65.5	122	54.4	369	8.65	3.22	749	31.6	59	8.85	31.1	7.73	1.37	5.42	1.23	9.21	2.19	6.58	1.36	4.64	1.24	8.13	0.891	67.8	7.96	3.18	
	13-20-2-30			66.5	149	50.5	317	10.6	3.39	860	29.1	67	8.04	35.9	8.4	1.42	9.65	1.2	7.07	1.41	5.26	0.851	5.13	0.87	8.69	0.809	40.4	7.87	2.71	
	13-20-2-31			65.7	136	46.2	296	10.1	3.2	670	26.7	57.5	7.5	32.1	8.17	1.07	7.36	1.43	7.5	1.65	3.92	0.738	5.14	0.952	7	0.516	39.3	7.09	2.48	
PCC-12 avg.		*All 20 um*	*72.17*	72.7	152	56.8	372	10.2	3.72	800	32.5	68.1	8.99	37.6	9.24	1.49	8.78	1.46	9.67	2.03	6.36	0.964	6.62	1.05	9.42	0.685	42.9	9.16	2.84	*24*
s.d.				6.75	19	7	44.8	0.855	0.38	78.2	3.68	5.95	0.895	4.92	2.17	0.348	2.66	0.244	1.73	0.334	1.25	0.248	1.42	0.23	1.71	0.173	6.48	1.49	0.411	
**PCC-14**	13-20-2-32	*20*	*72.17*	70.7	169	56.3	362	14.8	3.61	883	33.9	71.8	8.7	38.2	9.67	1.51	9	1.45	8.59	1.76	7.11	0.77	6.92	0.911	8.61	0.49	48	9.41	2.89	
	13-20-2-33			70.2	152	53.4	332	10.2	3.97	745	29.8	67.3	8.8	33.7	8.78	1.51	9.79	1.57	9.38	1.64	4.4	0.919	5.81	0.991	8.95	0.503	41.3	8.2	2.43	
	13-20-2-34			71.9	143	46.3	299	10.9	3.26	855	27.9	64.1	7.53	30.2	8.56	1.55	6.85	0.971	7.06	1.74	5.43	0.758	5.71	1.1	8.31	0.631	44.6	7.52	2.59	
	13-20-2-35			67.4	146	48	317	9.24	3.32	731	28	66	7.52	31.5	6.51	0.989	7.02	1.6	8.1	1.54	5.41	0.773	5.48	1.15	8.68	0.619	38.5	7.89	2.72	
	13-20-2-36			73.7	176	63.1	390	11.4	3.62	831	35.9	73.1	9.7	36.8	8.99	1.67	10.5	1.8	7.89	2.12	6.47	1.03	6.42	1.12	11.5	0.547	34.6	9.77	2.82	
	13-20-3-01			76.5	118	52.8	355	10.4	3.33	830	30.8	67.5	8.45	35.9	8.59	1.46	9.38	1.4	7.04	1.87	5.51	0.858	7.12	1.03	10.2	0.366	43.1	9.39	3.07	
	13-20-3-02			81.9	138	52	361	11.4	5.23	817	32.3	69.5	8.44	41.2	8.3	1.19	9.67	1.1	7.57	1.91	6.48	1.01	5.04	0.852	9.8	0.608	43	9.95	2.9	
	13-20-3-03			76.1	120	52.8	372	10.9	3.77	807	30.1	69.9	8.26	34.6	9.26	1.29	9.39	1.27	9.39	1.78	5.7	1.14	6.69	1.2	10.2	0.554	39.4	9.39	3.03	
	13-20-3-04			85.1	123	54.1	363	10	4.53	792	30.2	66.7	8.3	34.5	8.31	1.1	6.44	1.34	9.52	1.65	6.44	1.41	6.18	0.99	10.6	0.854	42.6	9.28	2.8	
	13-20-3-06			77.6	128	56.2	370	10.4	4.88	825	31.9	73.9	9.09	36.1	8.89	0.813	8.51	1.32	7.87	2.01	6.56	0.813	6.51	1.14	8.84	0.546	43	9.84	3.19	
	13-20-3-07			78.1	159	60.1	399	10.2	3.84	852	33.1	74.8	9.47	39.2	10.4	1.08	12.1	1.7	8.71	2.05	7.05	0.999	8.27	1.32	8.76	0.63	36.7	10.3	2.71	
	13-20-3-08			70.8	137	50.7	333	11.3	4.37	783	28.8	63.4	8.58	33.6	7.23	1.21	6.33	1.38	7.75	1.97	6.04	0.835	6.03	0.813	10.7	0.675	45	8.01	2.91	
	13-20-3-09			74.9	93.3	47.2	328	9	4.19	721	29.3	64.1	8.52	32.2	8.03	0.951	8.14	1.22	8.47	1.55	5.09	1.03	4.32	0.834	9.7	0.735	46.7	8.58	3.04	
	13-20-3-11			72.6	162	56.5	362	10.9	4.19	784	32.7	67.5	9.09	40.6	9.41	1.7	7.28	1.54	8.24	2.3	7.47	1.05	6.33	1.1	10	0.613	44.8	9.46	2.95	
	13-20-3-12			74.4	163	57.3	363	10.6	3.6	777	31.9	69.1	8.46	38.5	7.9	1.36	6.32	1.6	8.35	1.8	5.53	1.07	5.86	1.22	8.77	0.569	38.1	9.09	2.92	
	13-20-3-13			79.2	155	60.9	376	11.3	4.05	824	35.2	76.5	9.87	38.9	9.3	1.47	10.1	1.52	9.43	1.87	7.2	0.825	6.17	1.04	10.4	0.664	40.9	9.76	3.41	
	13-20-3-15			74.5	155	50.6	337	10.7	3.81	783	31.3	69.2	8.45	40.3	7.95	1.54	7.12	1.2	9.43	1.58	5.48	0.678	6.07	0.993	7.46	0.71	41.9	7.92	2.97	
	13-20-3-16			72.8	141	54.6	374	10.3	4.12	765	33.6	72.8	9.08	41.2	8.83	1.4	6.08	1.24	9.79	1.91	6.88	0.965	6.4	0.987	9.82	0.685	41.1	9.91	2.91	
	13-20-3-17			78.3	158	61.3	399	11.1	4.09	864	34.9	77.7	9.41	41	11.8	1.98	8.24	1.45	10.4	1.85	7.05	1.29	7.72	1.18	10.8	0.867	42.4	9.79	3.08	
	13-20-3-18			73.6	183	53.3	355	10.7	3.5	799	30.4	69.6	8.78	37.6	8.53	1.52	9.72	1.63	9.89	2.31	6.17	1.02	6.13	1.19	10.5	0.747	54.7	9.96	2.97	
	13-20-3-19			75.6	151	48.8	328	10.9	3.74	789	29.1	71.1	8.65	38.1	8.81	1.59	9.72	1.6	7.54	1.91	5.33	0.886	6.12	1.11	8.52	0.657	45.9	8.3	3.16	
	13-20-3-20			66.3	119	48	325	9.78	3.68	707	30	66.8	8.52	32.2	8.87	1.37	7.02	1.2	7.63	1.75	6.01	1.08	5.14	0.726	9.2	0.721	38.1	8	2.67	
	13-20-3-21			76.4	157	54.6	358	9.52	4.16	781	30.9	66.4	9.33	35.6	10.5	1.31	6.58	1.55	10.1	1.44	6.4	0.649	5.85	0.936	9.65	0.722	37.9	9.61	2.49	
	13-20-3-22			78.1	141	61.9	381	10.9	4.23	806	33.4	70.8	9.15	35.9	11.7	1.03	9.64	1.29	9.49	2.27	5.91	0.978	7.13	0.87	11.5	0.612	42.2	9.48	2.97	
	13-20-3-23			89.4	123	54.5	394	9.53	4.4	753	32.7	67.6	8.58	35.9	9.26	0.71	12	1.62	10.4	2.45	6.21	0.822	6.47	0.805	8.9	0.658	43.4	9.25	2.79	
	13-20-3-24			78.4	162	64.1	418	11.2	3.77	845	38.8	74.8	9.71	45.3	9.44	0.561	7.87	1.79	11.4	2.36	7.23	1.39	6.57	0.806	12.9	0.96	41.9	11.4	3.08	
	13-20-3-25			73.6	170	60	380	10.6	3.54	808	34.7	70.9	9.65	38.8	9.93	1.55	9.76	1.29	10	2.51	6.17	1.09	6.02	1.06	9.43	0.721	39.2	9.73	3.13	
	13-20-3-26			76	175	62.7	392	11.4	3.87	829	35.1	73.4	10.9	38.4	10.1	1.84	9.5	1.7	10.4	2.33	7.44	1.07	7.07	1.3	10.5	0.894	43.5	9.97	3.13	
	13-20-3-27			75.2	149	61.1	401	11.2	3.92	818	36	71.7	9.74	41.6	9.04	1.72	10	1.58	10.5	2.18	7.8	1.13	7.34	1.14	10.9	0.756	43.7	10.2	3.17	
PCC-12 avg.		*All 20 um*	*72.17*	75.5	147	55.3	363	10.7	3.95	800	32.2	69.9	8.92	37.2	9.06	1.34	8.62	1.44	8.98	1.95	6.27	0.977	6.31	1.03	9.79	0.666	42.3	9.29	2.93	*29*
s.d.				4.79	20.8	5.22	29	1.03	0.451	42.8	2.7	3.74	0.718	3.52	1.14	0.337	1.69	0.21	1.17	0.295	0.824	0.19	0.812	0.159	1.16	0.128	3.9	0.895	0.219	
**PCC-15**	13-20-4-01	*20*	*72.6*	81.9	139	64.2	432	11	5.6	977	37.4	84	11.2	44.1	10.7	1.72	9.68	1.74	9.96	2.36	7.5	1.24	7.52	1.27	11.5	0.755	54.7	10.7	3.16	
	13-20-4-02			79.7	123	55.6	370	10.1	4.43	784	33.4	69.3	8.6	34.8	9.18	1.14	9.26	1.36	9.57	1.98	5.68	0.911	6.83	1.04	10.2	0.694	47.2	9.87	3.32	
	13-20-4-03			80.7	125	57.3	375	10.5	4.3	816	32	69.4	9.41	39.6	9.61	0.985	7.4	1.38	7.98	1.81	5.45	1.17	6.49	0.915	10.5	0.527	46	9.12	2.8	
	13-20-4-04			78.2	156	56.5	370	10.9	3.74	799	32.3	69.5	8.46	37.1	8.04	1.23	9.69	1.37	10.4	1.89	6.39	1.16	7.18	0.893	10.4	0.721	45.4	10.1	3.09	
	13-20-4-07			85.1	169	74.5	483	11.6	4.12	850	39.2	73.1	10.6	54.2	8.93	1.8	10.3	2.01	14.4	2.43	4.32	1.47	7.35	1.27	14	0.469	42.8	10.5	2.68	
	13-20-4-09			81.6	142	68.7	468	10.3	3.86	872	37.6	75.5	10.5	46.2	10.2	2.16	10.7	1.7	9.86	2.59	6.1	1.29	7.67	1.38	13.6	0.615	41.9	11.8	2.96	
	13-20-4-10			79	129	57	389	9.31	4.32	836	34	72	8.75	40	10.6	1.45	10.7	1.52	8.72	1.85	5.99	0.82	6.83	1.04	11.1	0.6	49.5	10.8	3.18	
	13-20-4-11			80.1	125	58.8	398	10.7	4.23	854	33.2	74	9.46	37.2	9.05	1.57	10.9	1.31	9.53	2.8	6.08	0.954	7.73	1.22	10.5	0.775	50.2	10.5	3.4	
	13-20-4-12			82.9	166	67.1	449	11.2	3.7	911	38.6	75.2	9.26	40.1	10.7	1.75	8.74	1.77	9.54	1.94	8.59	1.21	8.66	1.33	13.3	0.692	44.9	11.7	2.71	
	13-20-4-13			85.1	153	62.8	414	9.68	3.92	856	36	71	9.42	43	9.49	2.18	10.2	1.26	12.4	2.51	6.61	0.955	6.83	1.15	9.56	1.16	44.1	9.53	3.15	
	13-20-4-14			84.9	132	59.6	403	10.9	4.29	832	34.5	72.7	9.55	40.1	9.18	1.51	8.34	1.59	11	2.31	8.12	0.862	6.75	1.02	10.2	0.969	48.1	10.7	3.3	
	13-20-4-15			80.9	124	55.3	388	10	4.23	788	31.3	71.1	8.89	39.1	7.12	1.36	8.27	1.5	7.9	2.35	6.33	1.18	6.72	1.08	10.8	0.983	45.3	9.39	3.12	
	13-20-4-16			81.7	124	55.4	369	11.5	4.54	800	31.9	74.1	9.12	38.6	8.65	1.26	9.28	1.48	8.5	2.09	6.45	0.92	6.61	1.05	10.1	0.706	44.7	9.7	3.44	
	13-20-4-17			81.4	138	54.5	354	10.8	4.12	790	33	70.1	9	37.8	7.81	1.56	8.5	1.36	9.58	2.14	5.21	1.02	5.77	0.958	9.36	0.626	47.4	9.53	3.07	
	13-20-4-18			90.2	115	56.1	393	11	4.8	791	32.7	72.7	9.32	37	7.97	1.01	7.09	1.26	9.44	1.66	6.57	0.924	7.05	1.08	11.1	0.811	52.3	10.2	3.27	
	13-20-4-19			78.9	164	56.6	354	11.2	3.76	750	31.4	66.5	9.11	37.9	8.62	1.52	7.7	1.48	10.1	1.6	6.68	0.956	7.04	0.957	8.83	0.697	46.1	9.66	3.44	
	13-20-4-20			85.8	121	54.9	370	10.6	4.57	750	31.2	71.1	8.6	36.5	8.79	1.02	7.79	1.34	9.45	2.07	6.33	1.02	5.71	1.07	9.01	0.625	44.9	8.82	3.3	
	13-20-4-21			83.5	124	58.3	371	10.7	3.82	780	33.9	72.7	9.15	34.5	10.3	1.3	9.61	1.4	9.28	2.25	6.43	1.03	6.81	0.967	10.3	0.715	45.9	9.87	3.41	
	13-20-4-22			85	135	60	408	10.5	4.52	815	33.3	71.7	9.74	40.4	7.65	1.32	8.63	1.75	9.32	1.95	7.27	0.687	7.84	1.03	10.7	0.607	48.5	9.79	2.91	
	13-20-4-23			84.1	163	67.5	438	10.1	4.93	914	39.1	82.1	10.4	40.7	10.4	2.08	9.7	1.66	8.77	2.26	7.97	1.26	8.07	1.06	12.8	0.67	47.1	10.9	2.87	
	13-20-4-24			79.1	133	60.5	398	10.4	4.44	806	34.2	70.7	9.26	38.1	7.84	1.34	10	1.43	9.96	2.1	5.79	0.911	7.43	1.23	10.3	0.771	46.4	10.1	3.05	
	13-20-4-25			84.4	133	61.4	393	11.1	4.35	846	35.5	74.3	9.65	38.1	9.7	1.51	8.66	1.48	8.96	2.36	6.58	1.15	7.43	1.18	10.7	0.772	44.9	9.81	3.21	
	13-20-4-26			79.9	158	54	361	10.9	4.27	780	31.9	65.4	8.79	39.3	11.3	1.46	8.21	1.45	9.02	2.13	6.96	1.36	6.3	1.16	9.21	0.748	55	8.89	2.69	
	13-20-4-27			82	151	62.7	408	9.73	4.23	829	34.8	73.8	9.41	37.7	9.19	1.09	12.2	1.66	10.6	2.19	7.18	1.12	7.34	1.12	10.2	0.616	44.7	9.67	2.99	
	13-20-4-28			80.9	131	57.9	392	10.5	4	803	34.4	67.1	8.33	42.4	10.3	1.15	10.7	1.44	8.67	1.81	5.32	1	6.96	0.757	10.9	0.945	43.8	9.36	3.06	
	13-20-4-29			85.8	141	69.2	455	11.3	4.06	863	38.7	74.7	9.89	44.4	9.61	1.78	9.73	1.45	10.2	2.42	8.48	1.46	7.19	1.29	12.3	0.669	50.2	11.1	3.01	
	13-20-4-30			79.8	141	58.6	375	12.2	4.09	784	33.1	70.1	8.46	39.3	8.78	1.19	10.4	1.43	8.01	1.85	6.62	0.822	6.53	0.999	10.8	0.434	44.8	9.24	3.35	
	13-20-4-31			79.9	136	56.3	380	11	4.25	797	33.2	70.3	8.88	36.5	10.5	1.41	10.3	1.49	9.8	1.84	6.23	0.932	6.9	0.938	11	0.712	43.8	9.34	3.34	
	13-20-4-32			80.1	138	55.6	373	11	4.39	781	32.5	70.4	8.51	37.9	8.47	1.64	7.73	1.25	8.42	2.25	6.4	1.05	5.56	0.968	9.44	0.673	45.5	8.84	2.84	
PCC-15 avg.		*All 20 um*	*72.60*	82.2	139	59.9	398	10.7	4.27	823	34.3	72.2	9.3	39.7	9.26	1.47	9.32	1.49	9.63	2.13	6.54	1.06	7	1.08	10.8	0.716	46.8	9.98	3.11	*29*
s.d.				2.78	15.1	5.24	34.1	0.612	0.394	50.8	2.47	3.91	0.7	3.9	1.08	0.328	1.24	0.178	1.33	0.289	0.968	0.193	0.687	0.145	1.31	0.153	3.23	0.789	0.232	
**PCC-16**	13-20-5-01	*20*	*72.45*	80.7	126	53.2	358	10.7	4.14	757	31.4	70.4	8.95	35.8	9.14	1.25	7.73	1.33	7.98	2.01	6.11	0.848	7.27	0.996	11.1	0.802	47	9.51	3.17	
	13-20-5-02			81.1	131	55.1	361	10.9	3.9	791	33.1	69.8	8.8	41.2	8.11	1.23	9.2	1.51	9.39	1.6	5.77	1.03	7.39	1.07	9.25	0.799	47.1	9.23	3.13	
	13-20-5-03			80.9	120	56.1	390	11	4.74	761	32.8	70	8.7	37.5	8.45	1.5	7.63	1.4	9.2	2.15	6.2	0.973	6.04	0.928	10.2	0.739	44.1	9.54	2.87	
	13-20-5-04			78.7	136	54.6	364	10.8	4.16	748	31.7	69.1	8.57	38.4	8.58	1.48	8.41	1.38	9.71	2.22	6.92	0.815	7.12	1.15	8.91	0.818	47.3	9.37	3.16	
	13-20-5-06			84	123	57.1	390	10.5	4.45	782	31.6	69.3	8.51	33.8	9.23	0.915	9.72	1.6	10.3	2.16	5.03	1.14	6.68	1.21	11.1	0.849	46.8	9.68	3.45	
	13-20-5-07			83.4	129	55.4	366	11	3.87	753	33.4	70.7	8.47	35.8	9.09	1.16	7.23	1.36	8.38	2.49	6.2	0.598	6.69	1.23	11.1	0.915	43.7	8.78	3.07	
	13-20-5-08			81.3	132	54.7	379	11.2	4.65	753	32.9	70.9	9.03	42.9	8.86	1.36	9.12	1.36	8.1	2.12	6.48	0.754	6.63	0.941	10.7	0.633	45.2	9.64	3.32	
	13-20-5-09			85.4	141	56.3	375	10.6	4.42	792	32	72.3	8.61	40.9	9.27	1.7	10.6	1.18	9.07	2.16	6.5	1.05	6.45	1.05	9.58	0.686	47.4	9.5	3.33	
	13-20-5-10			84.9	141	54.9	363	11.8	3.89	765	32	71.3	8.82	36	9.49	1.63	8.31	1.35	8.07	1.86	6.5	0.863	6.95	1.15	9.82	0.554	45.1	9.17	3.49	
	13-20-5-11			82.2	139	54.1	366	11.4	4.5	767	31.9	70.6	8.12	37.1	8.66	1.23	8.18	1.4	9.29	1.74	6.65	0.964	6	1.08	10.4	0.644	45.6	9.24	3.35	
	13-20-5-12			79.5	147	53.3	350	11.1	3.98	699	28.7	67.5	8.49	36.8	8.6	1.3	8.16	1.35	8.5	2.1	6.61	0.672	5.95	0.959	9.04	0.7	45	8.5	3.08	
	13-20-5-13			83.7	139	53.3	346	11.3	4.11	719	30	68.5	8.93	33.9	7.74	1.35	9.24	1.14	9.28	2.23	5.91	0.876	5.89	0.681	9.06	0.539	43.5	8.93	3.06	
	13-20-5-14			82.1	144	54.4	357	10.3	4.14	739	30.7	71.4	9.16	34.5	9.17	1.27	8.51	1.57	8.4	1.89	6.81	0.702	6.63	1.02	9.3	0.637	45.7	8.53	3.2	
	13-20-5-15			87.2	144	53.4	366	10.4	4.53	801	32.5	72.5	8.64	34.7	8.92	1.58	9.26	1.23	8.94	2.24	6.35	1.07	6.14	1.17	8.38	0.945	50.7	9.47	3.39	
	13-20-5-16			81.6	145	56.8	371	10.8	4.03	777	33.1	72.9	9.19	36.1	9.65	1.84	8.88	1.27	9.99	2.3	7.1	1.04	6.72	0.98	9.8	0.502	48.4	10.1	3.33	
	13-20-5-17			84.9	137	54	355	10.9	4.38	785	32	71	8.52	38	8.51	1.37	9.21	1.38	9.7	2.29	5.51	0.913	6.36	0.918	10	0.648	49.2	9.82	3.44	
	13-20-5-20			84.4	133	54.2	358	9.59	4.53	702	32.3	69.5	8.51	34.8	8.82	1.1	8.18	1.39	9.01	2.02	6.43	1.01	6.15	1.06	10.7	0.523	47	8.75	3.14	
	13-20-5-21			81.7	139	56.1	375	10.5	4.15	771	33.7	70.3	8.57	34.6	9.18	1.54	10.5	1.75	10.3	1.82	6.48	1.02	6.62	0.982	10.9	0.863	45.8	9.4	3.47	
	13-20-5-22			83.1	141	57.2	371	11.5	4.21	795	32.2	74.9	9.22	36.9	7.45	1.53	10.6	1.17	9.67	2.21	5.04	0.988	6.41	0.958	10	0.626	44.9	9.56	3.79	
	13-20-5-24			86.2	142	56.7	372	9.92	3.93	750	34.2	70	9.53	39.8	8.37	1.54	8.71	1.4	8.46	2.09	6.32	1.1	7.88	1.21	11	0.639	46	9.71	3.03	
	13-20-5-25			92.8	148	58.1	386	11.1	3.99	767	32.9	72.1	9.04	39.5	9.48	1.23	9.39	1.56	9.88	2.02	6.99	0.851	7.06	1.14	10	0.581	46.3	9.7	3.55	
	13-20-5-26			83.3	143	58.5	370	10.9	4.39	782	32.1	76	8.69	37.2	8.36	1.36	8.26	1.26	10.4	1.88	7.03	0.907	6.88	0.91	10.4	0.666	44.7	9.63	3.25	
	13-20-5-27			86.4	130	57.3	386	10.6	4.22	759	32.6	73.2	9	36.4	8.78	1.22	8.17	1.39	10.2	2.04	6.28	0.97	6.29	0.83	10.3	0.726	45.1	9.74	3.41	
	13-20-5-28			85.2	159	58.3	394	11.5	3.91	798	33.1	77.8	9.85	37.6	9.4	1.4	10.2	1.2	10.2	2.16	7	1.09	7.21	1.31	10.3	0.719	45.2	10.1	3.27	
	13-20-5-29			81.2	116	56.4	374	10	4.23	714	30.7	67	8.3	37.7	8.72	1.55	8.73	1.25	9.65	2.15	7.1	1.01	6.66	1.11	10.1	0.803	45.5	9.17	2.98	
	13-20-5-30			84.5	121	56.7	378	10.7	4.17	740	31.9	70.4	8.4	36.4	8.25	1.81	9.22	1.27	8.32	1.89	5.79	0.957	7.81	0.928	10.9	0.691	44	9.73	3.39	
	13-20-5-31			89.3	128	63	412	11.3	4.62	795	34.8	72.6	10	42	11.2	1.23	9.55	2.1	7.77	1.56	7.78	1.02	7.13	1.25	9.87	0.638	38	9.86	2.77	
	13-20-5-32			80.7	142	57.2	373	10.7	4.36	733	31.7	68.1	8.49	35.6	9.41	1.63	9.77	1.25	9.65	2.02	5.96	0.997	5.85	1.04	10.9	0.748	41.4	9.75	3.15	
	13-20-5-33			79.7	138	56.2	375	10.8	3.82	738	31.2	69.4	8.84	36.3	8.81	1.58	8.77	1.42	9.79	2.1	7.21	1.08	5.59	0.934	10.2	0.72	44.8	9.75	3.41	
PCC-16 avg.		*All 20 um*	*72.45*	83.5	136	55.9	372	10.8	4.22	760	32.2	71	8.83	37.2	8.89	1.41	8.95	1.39	9.22	2.05	6.42	0.941	6.64	1.04	10.1	0.702	45.5	9.44	3.26	*29*
s.d.				3.08	9.6	2.08	14.3	0.496	0.262	28.7	1.23	2.41	0.435	2.38	0.692	0.215	0.881	0.195	0.791	0.207	0.631	0.135	0.577	0.14	0.741	0.114	2.33	0.419	0.218	

**PCC-18**	13-20-6-01	*20*	*72.36*	82.2	157	61.9	419	12.4	4.14	869	37.9	75.2	10.7	45.4	8.74	1.51	8.74	1.76	11.2	2.62	7.75	1.03	7.89	0.929	12.5	0.679	45.3	10.6	3.18	
	13-20-6-02			80.6	144	58.2	394	10.7	4.3	775	31.9	70.2	8.38	41.3	9.63	1.54	11	1.54	7.8	2.31	7.04	0.807	6.54	1.08	10.5	0.703	40.6	9.2	2.91	
	13-20-6-03			84.3	138	56.9	380	11.7	4.23	770	33	70.5	8.8	36.4	8.15	1.43	9.99	1.23	9.53	2.41	6.53	1.17	6.81	1.06	9.5	0.666	39.5	9.53	2.9	
	13-20-6-04			79.1	136	55.2	378	11.1	3.91	751	33.3	69.6	8.71	32.4	8.72	1.35	10.5	1.85	9.54	1.89	6.28	0.808	6.68	1.13	9.61	0.648	46.1	10.1	3.29	
	13-20-6-05			83.3	175	66.5	425	11.8	4.99	847	37	83.1	10.5	41.5	10.7	1.83	10	1.55	12.1	2.71	6.2	1.03	8.13	0.944	10.7	0.907	36.4	10.5	3.32	
	13-20-6-06			74.5	164	59.5	379	10.7	4.12	765	34.5	69.9	9.35	38.8	9.69	1.59	6.87	1.48	10.9	2.08	7.62	1.29	7.17	1.2	10.7	0.56	42	9.61	2.83	
	13-20-6-07			84.7	129	62.8	415	10.6	4.35	802	34.2	71.1	9.55	38.1	10.8	1.71	7.86	1.42	11.1	2.32	7.32	1.36	7.01	1.07	12.3	1.16	43.5	9.96	2.77	
	13-20-6-08			83.1	126	57.6	404	10.2	3.95	758	34.4	74.8	9.1	35.6	7.97	1.39	6.91	1.93	8.7	2.45	6.59	0.894	7.83	0.888	10.7	0.807	45.5	10.5	3.41	
	13-20-6-09			82	143	55.3	390	10.9	4.21	758	31.9	69	9.1	33	8.82	1.11	9.85	1.25	9.69	2.18	6.51	0.726	6.86	1.22	10.4	0.719	46.3	9.95	3.28	
	13-20-6-10			82.4	122	60.3	406	11.1	4.81	778	33.8	73.7	9.13	41.5	8.8	1.66	9.96	1.74	8.59	2.44	7.03	1.06	7.28	1.17	10.9	0.67	43.7	9.48	3.36	
	13-20-6-11			80.7	166	69.3	471	11	4.04	855	38.3	74.8	10.6	42	8.62	2.51	14.1	1.57	12.6	2.38	8.83	1.36	8.94	1.21	13.5	0.828	37.1	10.5	2.86	
	13-20-6-12			87	160	61.4	403	11.1	5.04	858	37.5	78.1	10.4	42.6	10.4	1.7	10.7	1.54	8.04	2.47	7.99	0.856	6.63	1.12	11.2	0.584	45.2	10.9	3.4	
	13-20-6-13			84.9	143	58.5	396	11.1	4.27	744	34	73.3	8.96	38.8	8.03	1.59	8.31	1.64	8.64	2.33	6.59	0.963	7.55	1.25	10.6	0.658	42.7	9.22	3.32	
	13-20-6-15			83.5	163	70.5	392	11	4.16	810	40.8	88.3	11	56.8	12.4	1.46	10.2	1.84	10.6	2.95	8.52	0.919	7.28	1.22	10.6	0.858	46	11	3.53	
	13-20-6-16			83	141	67.6	418	11.2	3.91	844	39.3	75.5	10.2	46.7	8.78	1.78	15.9	1.38	8.61	2.53	6.8	0.785	7.04	0.929	12.2	0.689	41.8	10.6	3.53	
	13-20-6-17			83.6	146	62	412	11.3	4.49	793	34.6	72.6	9.89	40.3	11.3	1.86	10.7	1.59	9.89	2.15	6.77	1.23	7.21	1.65	10.8	0.587	45.2	10.8	3.36	
	13-20-6-18			83.3	142	60.7	383	11.4	4.25	762	34.2	72.1	9.25	39.8	9.76	1.5	8.68	1.57	9.01	2.09	7.78	0.92	7.41	0.977	9.39	0.927	46.6	10.1	3.3	
	13-20-6-20			77.5	163	74.6	452	11.2	3.67	809	38.2	70.1	10.5	46.7	9.98	2.21	10.9	1.87	10	3.43	9.15	0.948	9.12	1.36	12.8	0.538	41.7	12.1	3.24	
	13-20-6-21			86.3	162	79.3	488	10.9	3.51	862	43	72.6	10.7	50.8	11.1	1.71	12.5	2.1	11.6	2.16	6.01	2.26	6.41	0.766	12.4	0.614	38.2	9.61	2.54	
	13-20-6-25			79.8	173	79.2	491	10.8	3.9	872	42.5	81	10.9	48.6	9.37	2.06	11.6	2.31	10.2	3.36	8.97	1.32	9.48	1.12	12	0.746	41.7	12.7	3.38	
	13-20-6-26			79.9	147	58.5	404	11	4.11	775	35	70.9	9.2	42.1	10	1.6	9.86	1.65	7.86	2.33	7.27	1.26	6.23	1.21	11.7	0.623	44.2	10.6	3.19	
	13-20-6-27			85.4	147	73.6	498	13	4.26	893	41.5	80.1	10.9	51	11.8	1.6	11.6	1.97	12.5	2.97	8.91	1.08	8.67	1.19	11.5	0.784	45.2	12.3	3.12	
	13-20-6-28			76	147	58.5	412	11.5	3.78	752	35.9	69.5	9.51	42.2	9.75	1.02	11	1.43	9.78	1.55	7	0.866	6.96	1.06	10.2	0.585	64.8	9.94	2.62	
	13-20-6-29			81.2	125	61.3	435	11.5	4.26	806	35.5	75.4	9.25	39.4	8.7	0.989	11.3	2.01	9.06	2.38	5.63	1.02	7.89	1.21	12.3	0.649	43.6	10.8	3	
	13-20-6-30			81.8	129	63.4	425	10.7	4.03	800	34.8	71.7	9.61	37.1	9.88	1.49	11.1	1.55	10.2	2.38	6.37	1.04	7.71	1.18	11.1	0.798	40.9	10.8	3.11	
	13-20-6-31			82.2	136	64.4	453	11.3	4.18	797	35.5	73.2	10	37.2	10.8	1.78	10.1	1.65	10.6	2.06	7.95	1.12	8.08	1.37	11.8	0.702	41.7	11	3.07	
	13-20-6-32			80.8	147	62.9	436	11.2	4.14	780	37.2	74.7	9.9	40.1	9.18	1.47	11.3	1.57	10.1	2.81	7.64	1.16	7.59	0.984	12.5	0.618	43.1	10.2	2.99	
	13-20-6-33			81.9	150	63.2	415	11	4.18	784	33	71.6	9.27	45.2	9.77	0.983	11.3	1.86	8.54	2.03	6.69	1.09	7.63	1.26	13	0.697	40.6	10.6	3.07	
PCC-18 avg.		*All 20 um*	*72.36*	82	147	63.7	421	11.2	4.19	803	36.2	74	9.76	41.8	9.7	1.59	10.5	1.67	9.9	2.42	7.28	1.08	7.5	1.13	11.3	0.714	43.5	10.5	3.14	*28*
s.d.				2.87	14.7	6.65	34	0.561	0.342	43.2	3.08	4.55	0.758	5.56	1.14	0.342	1.9	0.253	1.35	0.411	0.964	0.293	0.824	0.175	1.1	0.134	5	0.852	0.262	
**PCC-7**	13-19-4-01	*20*	*72.2*	74.4	176	60.8	385	10.5	3.9	750	34.3	69.1	9.39	39.8	8.71	1.69	10.8	1.81	10.4	2.39	7.42	1.16	7.53	1.01	9.82	0.73	42.1	10.6	2.83	
	13-19-4-02			76.5	147	61.1	401	10.5	3.79	723	32	71.5	8.78	41.5	9.36	1.21	9.11	1.41	9.81	2.08	6.18	0.997	7.39	0.995	9.65	0.611	43	10.2	3.33	
	13-19-4-03			75.7	163	55.3	360	10.5	3.87	734	29.5	66.9	8.77	38.4	8.46	1.39	7.58	1.57	9.01	2.03	7.07	0.989	6.02	0.9	9.69	0.608	44.9	9.6	3.24	
	13-19-4-04			81.6	132	61.9	411	12.2	4.1	748	35.2	69.4	8.95	42.1	7.87	1.23	9.04	1.41	9.37	2.66	6.23	1.05	6.38	0.784	10.5	0.739	45.1	11	2.8	
	13-19-4-05			78.2	129	58	398	10.5	4.04	752	33.4	68.7	8.9	37.6	9.18	1.36	8.99	1.54	10.1	2.07	7.14	1	6.16	0.925	11	0.701	40.9	10.4	2.98	
	13-19-4-06			74.4	141	58.5	384	10.2	3.87	706	32	66.6	9.42	38.4	8.72	1.37	8.97	1.32	9.89	2.41	5.84	0.859	5.52	0.87	11.2	0.782	41.1	9.58	3.04	
	13-19-4-07			75.4	143	60.7	390	11.2	3.72	743	32.9	69.6	9.24	39.7	8.57	1.67	9.18	1.47	9.64	2.25	6.82	0.746	7.32	0.99	10.3	0.634	41	9.81	2.98	
	13-19-4-08			74.6	137	59.7	376	10.4	3.81	717	31.9	70.1	8.72	39.7	8.32	1.45	9.13	1.61	10.2	2.08	6.43	1.1	6.79	1.02	10.6	0.871	42.4	9.68	3.08	
	13-19-4-09			78	153	61.7	413	11.9	4.39	799	35.8	74.5	9.32	40.8	9.67	2.02	9.99	1.61	10.9	2.29	7.44	1.03	7.68	1.17	12	0.825	44.3	10.9	3.53	
	13-19-4-10			74.7	186	66.6	432	10.7	3.89	802	37.9	75.3	10.1	44.6	9.85	1.02	12.1	1.81	9.51	2.43	6.99	1.01	7.78	1.36	12.2	0.858	41	10.5	3.17	
	13-19-4-12			76.3	156	66.6	417	12.6	3.89	829	37	69.5	9.29	42.7	8.92	1.61	10.4	1.61	10.8	2.39	7.16	1.01	8.28	1.02	11.2	0.747	42	10.3	2.84	
	13-19-4-13			76.7	153	62.7	417	12.3	4.33	775	33.5	70.6	8.94	43.2	8.22	1.9	10.4	1.65	11.4	2.36	7.4	1.14	7.75	1.25	10.6	0.643	41.6	10.4	3.22	
	13-19-4-14			72.9	155	63.4	414	11	3.67	785	34.8	71.5	9.04	45.2	10.1	1.33	8.26	1.85	10.7	2.31	6.49	0.964	7.35	1.14	10.7	0.805	43.1	11.2	3.23	
	13-19-4-15			77.6	147	62.6	403	11.1	3.98	752	35.8	70.6	8.95	43.1	9.46	1.65	9.8	1.62	10.7	2.27	7.5	1.06	7.17	1.23	10.6	0.798	42.6	10.7	3.04	
	13-19-4-16			73.6	148	61.9	415	11.5	4.2	769	35	70.4	9.16	40.8	8.38	1.2	8.25	1.77	10.7	2.19	7.15	1.26	7.71	1.19	10.6	0.704	43.1	10.5	3.2	
	13-19-4-17			79.6	148	61.7	402	11.2	3.84	804	34.9	72.8	9.37	40.9	8.37	1.39	9.53	1.59	10.3	2.44	7.3	0.834	7.71	1.21	10.6	0.74	44.2	10.3	3.41	
	13-19-4-18			76.9	149	63.6	417	11.3	4.07	752	33.1	73.2	9.53	40.7	8.28	1.53	11.4	1.93	11	2.13	7.86	1.16	8.07	1.26	10.7	0.753	41.1	11.3	3.21	
	13-19-4-19			76	152	60.8	416	11.7	3.79	791	34.4	73.7	9.94	45.3	11.3	1.08	10.4	1.48	11.4	2.58	6.44	1.31	7.9	1.24	10.7	0.661	45	10.7	3.39	
	13-19-4-20			75.4	154	62.8	427	11.4	4.22	814	35.8	71.9	10.2	40.7	9.06	2.09	11	1.78	10.4	2.45	7.35	1.29	8.21	1.31	12.5	0.909	43.1	12.1	3.33	
	13-19-4-22			72.3	167	61.9	389	11	3.93	756	33.8	69.9	9.84	42.2	9.51	1.71	7.8	1.49	10.4	2.09	7.22	1.27	7.3	1.31	10.5	0.796	42	10	3.23	
	13-19-4-23			78.9	151	60	430	11.1	4.01	833	37.1	74.4	10.4	40.8	9.96	1.97	13.7	1.97	9.6	2.31	5.56	1.14	7.96	1.44	11.2	0.848	46.8	11.6	3.24	
	13-19-4-24			76.1	126	62.5	416	10.3	4.09	737	34	72.9	8.59	41.8	9.42	1.16	11.3	1.55	9.72	2.79	7.05	0.935	7.96	1.55	10.5	0.774	39.7	10.4	2.84	
	13-19-4-25			78	143	58.3	393	11.4	4.26	745	34.2	71.3	9.23	39.9	7.65	1.78	9.18	1.59	10.5	2.41	7.85	1.25	6.88	1.16	12.2	0.73	43.6	10.7	3.26	
PCC-7 avg.		*All 20 um*	*72.20*	76.3	150	61.4	405	11.2	3.99	766	34.3	71.1	9.3	41.3	9.01	1.51	9.84	1.63	10.3	2.32	6.95	1.07	7.34	1.15	10.8	0.751	42.8	10.5	3.15	*23*
s.d.				2.2	14	2.54	18.2	0.664	0.196	35.4	1.92	2.3	0.494	2.07	0.839	0.308	1.45	0.171	0.64	0.197	0.608	0.151	0.738	0.192	0.766	0.0837	1.7	0.622	0.201	
																													**Total**	**317**

		Crater dia.	CaO (wt%)	**Rb**	**Sr**	**Y**	**Zr**	**Nb**	**Cs**	**Ba**	**La**	**Ce**	**Pr**	**Nd**	**Sm**	**Eu**	**Gd**	**Tb**	**Dy**	**Ho**	**Er**	**Tm**	**Yb**	**Lu**	**Hf**	**Ta**	**Pb**	**Th**	**U**	
**ATHO-G GeoReM**	Avg.		1.7	65.3	94.1	94.5	512	62.4	1.08	547	55.6	121	14.6	60.9	14.2	2.76	15.3	2.51	16.2	3.43	10.3	1.52	10.5	1.54	13.7	3.9	5.67	7.4	2.37	
**Accepted Values**	95% C.L.			3	2.7	3.5	20	2.6	0.11	16	1.5	4	0.4	2	0.4	0.1	0.7	0.08	0.7	0.11	0.5	0.07	0.4	0.05	0.5	0.2	0.62	0.27	0.12	
**ATHO-G May 2014**		*20 um*	1.66	65.1	94.6	99.6	524	62.9	1.01	529	54.7	118	14.1	59.6	14.1	2.69	15.7	2.62	16.3	3.69	11.1	1.58	10.6	1.55	14.1	4.02	6.22	7.31	2.38	*36*
**Analyses**			0.17	3.45	5.16	4.72	28.04	2.29	0.13	22.56	2.56	7.8	0.73	4	1.3	0.3	1.36	0.27	1.18	0.27	1.19	0.15	0.9	0.19	1.06	0.23	1.51	0.47	0.2	

SI Table 4: Bulk glass shard major (XRF) & trace (Solution-ICP-MS) element compositions of Puyehue-Cordón Caulle (PCC) tephra erupted June 4th to 14th 2011

	PCC-1	PCC-2	PCC-4	PCC-6	PCC-8	PCC-10	PCC-11	PCC-12	PCC-14	PCC-15	PCC-16	PCC-18	PCC-7 a	PCC-7 b	PCC-19	PCC-20	BCR-2	BHVO-2	BCR-2	BHVO-2
	4th June 2011			6th June 2011				8th June 2011					14th June 2011				Measured standards		GeoReM recommended values	
SiO_2	62.71	63.64	63.78	68.16	65.42	67.63	68.95	67.75	68.36	68.39	64.20	69.21	67.62	67.55	69.14	68.40	54.05	49.76	54.1 (± 0.80)	49.9 (± 0.60)
TiO_2	1.08	1.03	1.00	0.53	0.95	0.90	0.66	0.71	0.68	0.73	1.22	0.60	0.84	0.84	0.70	0.66	2.26	2.74	2.26 (± 0.05)	2.73 (± 0.04)
Al_2O_3	15.37	15.64	15.23	13.50	15.00	14.31	14.03	14.44	14.13	14.53	14.94	13.91	14.28	14.28	14.11	13.98	13.53	13.60	13.5 (± 0.20)	13.5 (± 0.20)
FeO*	6.29	5.80	5.89	3.49	5.36	4.84	4.02	4.32	4.12	4.34	6.43	3.77	4.68	4.63	4.14	3.98	13.84	12.44	13.8 (± 0.20)	12.3 (± 0.20)
MnO	0.16	0.15	0.14	0.10	0.14	0.13	0.11	0.12	0.12	0.12	0.16	0.11	0.13	0.13	0.12	0.12	0.18	0.16		
MgO	1.84	1.60	1.72	0.50	1.31	0.86	0.64	0.86	0.70	0.79	1.65	0.55	0.84	0.83	0.65	0.63	3.68	7.40	3.59 (± 0.05)	7.23 (± 0.12)
CaO	4.54	4.25	4.22	1.87	3.48	2.52	2.13	2.65	2.25	2.56	3.78	1.96	2.49	2.46	2.17	2.13	7.15	11.51	7.12 (± 0.11)	11.40 (± 0.20)
Na_2O	4.77	4.99	4.77	5.22	5.08	5.10	5.17	5.12	5.18	5.33	4.87	5.18	5.16	5.11	5.17	5.27	3.15	2.20	3.16 (± 0.11)	2.22 (± 0.08)
K_2O	1.89	1.97	2.06	2.79	2.24	2.61	2.80	2.59	2.71	2.60	2.13	2.82	2.59	2.58	2.76	2.74	1.82	0.52	1.79 (± 0.05)	0.52 (± 0.01)
P_2O_5	0.23	0.21	0.21	0.13	0.19	0.16	0.14	0.16	0.15	0.15	0.20	0.14	0.16	0.16	0.14	0.15	0.38	0.31	0.35 (± 0.02)	0.27 (± 0.02)
LOI	0.39	0.11	0.44	3.27	0.33	0.55	0.82	0.81	0.91	0.05	-0.18	1.30	0.86	1.13	0.28	1.55	0.07	-0.43		
Total	99.28	99.38	99.45	99.57	99.51	99.60	99.49	99.54	99.31	99.60	99.40	99.56	99.64	99.70	99.36	99.61	100.11	100.21		

Results are expressed as wt % on an oven dried 110°C basis
LOI = loss on ignition at 1000°C for 1 hour

	PCC-1	PCC-2	PCC-4	PCC-6	PCC-8	PCC-10	PCC-11	PCC-12	PCC-14	PCC-15	PCC-16	PCC-18	PCC-7 a	PCC-7 b	PCC-19	PCC-20	BCR-2 a	BCR-2 b	BCR-2
																	Measured standards		GeoReM recommended values
^{7}Li	23.17	30.10	26.11	41.72	27.55	30.75	32.73	30.65	33.68	31.25	26.44	32.69	31.48	30.69	31.50	31.34	9.64	9.71	9
^{45}Sc	24.56	21.43	21.16	11.91	19.52	15.32	13.41	15.29	14.29	14.97	21.39	12.32	15.52	15.24	13.60	13.51	31.8	32.7	33
^{49}Ti	6090.22	6089.59	5933.43	3143.08	5660.44	5377.26	4083.23	4273.39	4148.36	4390.69	7289.00	3552.25	5127.65	5028.29	4244.19	3980.33	21600	21700	22600
^{51}V	65.16	52.40	74.91	8.87	37.25	28.70	16.97	24.14	13.87	17.51	62.39	12.01	20.31	21.31	16.36	12.56	398	399	416
^{53}Cr	9.36	5.48	13.06	1.32	3.04	2.23	1.77	2.89	0.59	0.90	8.06	1.22	0.62	0.64	1.11	0.52	12.6	13.2	18
^{59}Co	9.97	8.76	10.12	2.92	7.19	5.56	4.09	5.01	4.00	4.52	9.70	3.32	5.04	5.05	4.07	3.70	35.7	35.9	37
^{60}Ni	3.86	3.52	5.14	0.55	2.09	1.20	0.96	1.69	0.43	0.53	4.15	0.61	0.63	0.60	0.76	0.42	4.05	8.44	18
^{63}Cu	38.35	30.46	40.02	18.22	27.48	26.49	21.90	24.24	19.59	19.55	33.29	19.02	24.63	23.32	21.60	19.69	13.6	15.9	21
^{66}Zn	95.95	90.75	90.68	75.78	90.67	90.75	82.72	83.69	83.98	86.30	103.11	77.79	88.67	88.95	83.18	81.52	124	131	127
^{71}Ga	19.91	20.69	20.45	19.44	20.37	20.40	20.22	20.32	20.05	20.68	20.59	19.48	20.38	20.14	20.06	20.01	21.5	22.1	23
^{85}Rb	47.74	50.11	53.33	74.16	57.14	69.21	74.05	68.18	71.59	69.17	55.52	74.38	68.01	67.99	73.31	72.93	45.8	46.5	46.9
^{86}Sr	292.38	299.04	270.58	137.19	242.60	185.43	158.80	193.21	169.89	192.11	251.86	143.17	187.00	182.68	162.27	162.48	326	340	340
^{89}Y	44.04	43.92	45.59	54.98	48.52	53.45	55.63	53.17	54.92	53.58	47.61	54.54	53.19	52.75	54.92	54.64	35	35.6	37
^{91}Zr	227.03	235.95	251.36	349.13	272.79	326.78	353.21	326.27	337.81	324.59	261.51	348.73	323.73	319.45	345.84	347.55	174	178	184
^{93}Nb	7.49	7.81	8.06	9.45	8.77	9.91	9.96	9.32	9.53	9.34	8.93	9.50	10.02	9.41	9.93	9.73	11.6	11.9	12.6
^{95}Mo	1.50	1.62	1.75	2.30	1.90	2.16	2.28	2.09	2.16	2.18	1.82	2.22	2.15	2.12	2.23	2.19	282	287	250
^{133}Cs	4.19	4.44	4.49	6.16	4.74	5.74	6.12	5.64	5.89	5.68	4.60	6.07	5.68	5.61	5.94	5.93	1.15	1.14	1.1
^{137}Ba	559.62	584.40	600.22	743.68	629.16	719.42	756.11	712.71	728.72	719.33	605.02	726.59	709.09	701.93	726.65	728.81	668	684	677
^{139}La	23.12	24.57	24.73	31.62	26.05	29.68	31.56	29.17	30.20	30.26	25.58	31.08	29.76	29.20	31.14	30.86	24.5	24.9	24.9
^{140}Ce	52.57	55.53	55.80	70.76	59.47	67.58	70.64	67.25	67.82	67.44	57.38	68.65	68.41	65.60	70.36	71.06	50.5	51.1	52.9
^{141}Pr	7.00	7.21	7.42	9.10	7.70	8.72	9.22	8.67	8.75	8.75	7.68	9.15	8.74	8.65	8.99	8.96	6.71	6.78	6.7
^{146}Nd	30.13	30.99	31.71	37.42	32.77	36.52	37.86	36.45	36.83	36.36	32.13	36.93	36.17	35.85	37.69	37.60	28.5	28.8	28.7
^{147}Sm	6.91	7.04	7.33	8.66	7.63	8.42	8.61	8.24	8.37	8.20	7.32	8.28	8.49	7.99	8.56	8.49	6.5	6.47	6.58
^{151}Eu	1.91	2.02	1.84	1.60	1.89	1.78	1.69	1.79	1.74	1.85	1.87	1.66	1.79	1.77	1.77	1.73	2	1.99	1.96
^{157}Gd	7.16	7.26	7.59	8.68	7.87	8.53	8.65	8.33	8.44	8.43	7.59	8.50	8.48	8.35	8.62	8.49	6.85	6.78	6.75
^{159}Tb	1.15	1.15	1.19	1.38	1.25	1.36	1.39	1.33	1.36	1.37	1.22	1.38	1.35	1.33	1.38	1.39	1.03	1.03	1.07
^{163}Dy	7.49	7.49	7.81	9.03	7.97	8.89	9.16	8.68	8.90	8.72	7.78	8.83	8.86	8.52	8.78	8.81	6.36	6.35	6.41
^{165}Ho	1.56	1.60	1.64	1.91	1.67	1.87	1.94	1.85	1.89	1.87	1.66	1.90	1.86	1.84	1.92	1.90	1.28	1.28	1.28
^{166}Er	4.56	4.63	4.84	5.81	5.01	5.62	5.83	5.55	5.67	5.62	4.92	5.74	5.58	5.50	5.80	5.77	3.61	3.58	3.66
^{169}Tm	0.69	0.69	0.72	0.88	0.76	0.86	0.89	0.85	0.86	0.85	0.75	0.88	0.85	0.84	0.87	0.86	0.51	0.52	0.54
^{172}Yb	4.59	4.68	4.82	5.87	5.06	5.76	6.04	5.68	5.82	5.77	4.98	5.93	5.69	5.60	5.86	5.86	3.3	3.31	3.38
^{175}Lu	0.70	0.71	0.77	0.95	0.80	0.87	0.91	0.86	0.88	0.86	0.74	0.89	0.91	0.84	0.89	0.89	0.48	0.48	0.5
^{178}Hf	5.78	6.03	6.51	8.96	6.94	8.43	8.99	8.33	8.66	8.39	6.82	8.87	8.37	8.23	8.81	8.73	4.56	4.55	4.9
^{181}Ta	0.44	0.45	0.46	0.62	0.49	0.62	0.58	0.55	0.58	0.55	0.50	0.56	0.57	0.56	0.57	0.56	0.74	0.75	0.74
^{182}W	0.24	0.24	0.30	0.39	0.31	0.40	0.42	0.40	0.40	0.44	0.36	0.45	0.36	0.42	0.45	0.45	0.33	0.33	
^{208}Pb	20.11	19.17	20.57	31.18	21.53	25.77	27.16	25.38	26.07	26.08	21.13	27.47	25.44	25.69	26.22	26.26	12.9	13.2	11
^{232}Th	5.76	5.96	6.57	8.98	6.88	8.57	9.08	8.35	8.59	8.37	6.83	9.04	8.29	8.20	8.78	8.76	5.64	5.47	5.7
^{238}U	1.54	1.60	1.75	2.40	1.82	2.28	2.44	2.24	2.29	2.23	1.78	2.39	2.20	2.17	2.20	2.19	1.48	1.51	1.69
